# Dual sensing signal decoupling based on tellurium anisotropy for VR interaction and neuro-reflex system application

**DOI:** 10.1038/s41467-022-33716-9

**Published:** 2022-10-10

**Authors:** Linlin Li, Shufang Zhao, Wenhao Ran, Zhexin Li, Yongxu Yan, Bowen Zhong, Zheng Lou, Lili Wang, Guozhen Shen

**Affiliations:** 1 State Key Laboratory for Superlattices and Microstructures, Institute of Semiconductors, Chinese Academy of Sciences, Beijing, 100083 China; 2 Center of Materials Science and Optoelectronic Engineering, University of Chinese Academy of Sciences, Beijing, 100083 China; 3 School of Integrated Circuits and Electronics, Beijing Institute of Technology, Beijing, 100081 China

**Keywords:** Electronic devices, Electronic devices

## Abstract

Anisotropy control of the electronic structure in inorganic semiconductors is an important step in developing devices endowed with multi-function. Here, we demonstrate that the intrinsic anisotropy of tellurium nanowires can be used to modulate the electronic structure and piezoelectric polarization and decouple pressure and temperature difference signals, and realize VR interaction and neuro-reflex applications. The architecture design of the device combined with self-locking effect can eliminate dependence on displacement, enabling a single device to determine the hardness and thermal conductivity of materials through a simple touch. We used a bimodal Te-based sensor to develop a wearable glove for endowing real objects to the virtual world, which greatly improves VR somatosensory feedback. In addition, we successfully achieved stimulus recognition and neural-reflex in a rabbit sciatic nerve model by integrating the sensor signals using a deep learning technique. In view of in-/ex-vivo feasibility, the bimodal Te-based sensor would be considered a novel sensing platform for a wide range application of metaverse, AI robot, and electronic medicine.

## Abstract (web summary)

The accumulation of single-function sensors can increase the complexity of virtual reality systems. Here, Shen et al. exploit the intrinsic anisotropy of tellurium nanowires to design a multi-function pressure and temperature sensor, which can be used as tactile experience in the virtual world.

## Introduction

Tellurium (Te) is an emerging semiconductor material for next-generation devices with low thermal conductivity (1.6 W m^−1^ K^−1^)^1,2^, excellent transport properties^3,4^, high carrier mobility of ~1000 cm^2^V^−1^s^−1^ ^5,6^, and anisotropic atomic structure^7,8^. These properties make Te a promising candidate as the material of choice for next generation electronic and photonic devices^9–11^. Currently, the trend of modernizing flexible electronics increasingly requires convenience and multifunctionality for future devices^12–15^. In recent years, research effort in electronic skin has continued to increase, which is reflected in the successful demonstration of many bimodal sensors for human-machine interaction^16^, neuromorphic Computing^17^, metaverse^18^, and medical healthcare applications^19^. Zhu et al. successfully prepared a bionic multidimensional sensor for a manipulator that can detect a variety of signals and improve the accuracy of recognition compared with a single signal^20^, and Chen et al. combined vision and stretch sensors to achieve human-computer interaction (HMI)^21^. These two studies attempted to integrate different types of sensors, but the developed systems were structurally complex owing to the one-to-one (OTO) detection mode. Therefore, multimodality in a single sensing unit by adopting the one-to-multitude (OTM) mode, could be an effective strategy to simplify the structure of the system. To date, examples of OTM mode devices are limited because of the key requirements of the versatility of the materials and the unique architecture design of the devices. A single-crystal Te semiconductor is composed of ternary helical chains of Te atoms, which are combined into a hexagonal system under the action of van der Waals forces^22,23^. In this structure, the relatively heavy atomic mass and large phonon scattering between molecular chains result in a low lattice thermal conductivity^24^. Te exhibits good thermoelectric and electrical conductivity along the z axis^2^. More importantly, Te with an intrinsic anisotropic atomic structure can modulate electron directional polarization to realize signal separation of different stimuli and can intrinsically minimize signal interference. Hence, it is feasible to develop state-of-the-art multifunction sensing devices using Te semiconductors.

Here, the feasibility of simultaneous measurement of pressure and temperature difference signals is demonstrated through the orientation design of an external stimulus using the anisotropy of a Te electronic structure to control electron directional polarization. We present a biocompatible bimodal tactile sensor (BTS) combined with a vertical array of Te NWs that can differentiate between thermal and pressure signals without signal interference. Two variables were derived from the IV characteristic curve of the tactile sensor, namely voltage as a stress-insensitive variable to measure the temperature difference and resistance as a temperature-insensitive variable, within a small range, to measure stress. The tactile sensor simultaneously detects the temperature difference and stress by measuring the current at two different bias voltages, which eliminates the difficulty of voltage and resistance simultaneous measurement in the circuit. Meanwhile, the self-locking effect in the device structure design can eliminate the dependence of the tactile sensor on displacement. A smart gloves prepared by Te-BTS can learn to classify based on the hardness and thermal conductivity of the material by integrating the sensor signals using a deep learning technique. This system can not only capture the interaction between human actions and the environment, but also map the recognition results of the material and hardness of the actual object in reality to the virtual space, so as to improve the somatosensory feedback of VR. This is attempt to enriching the interaction between Virtual Reality (VR) and physical world. In addition, we demonstrated, through an in vivo test, that a neural reflex circuit based on this sensor responds to the hardness and thermal conductivity of the material selectively, similar to adaptive mechanoreceptors in human skin, and can generate sensory neuron-like output signal patterns.

## Results

### Structure of tellurium

**Fig. 1:**
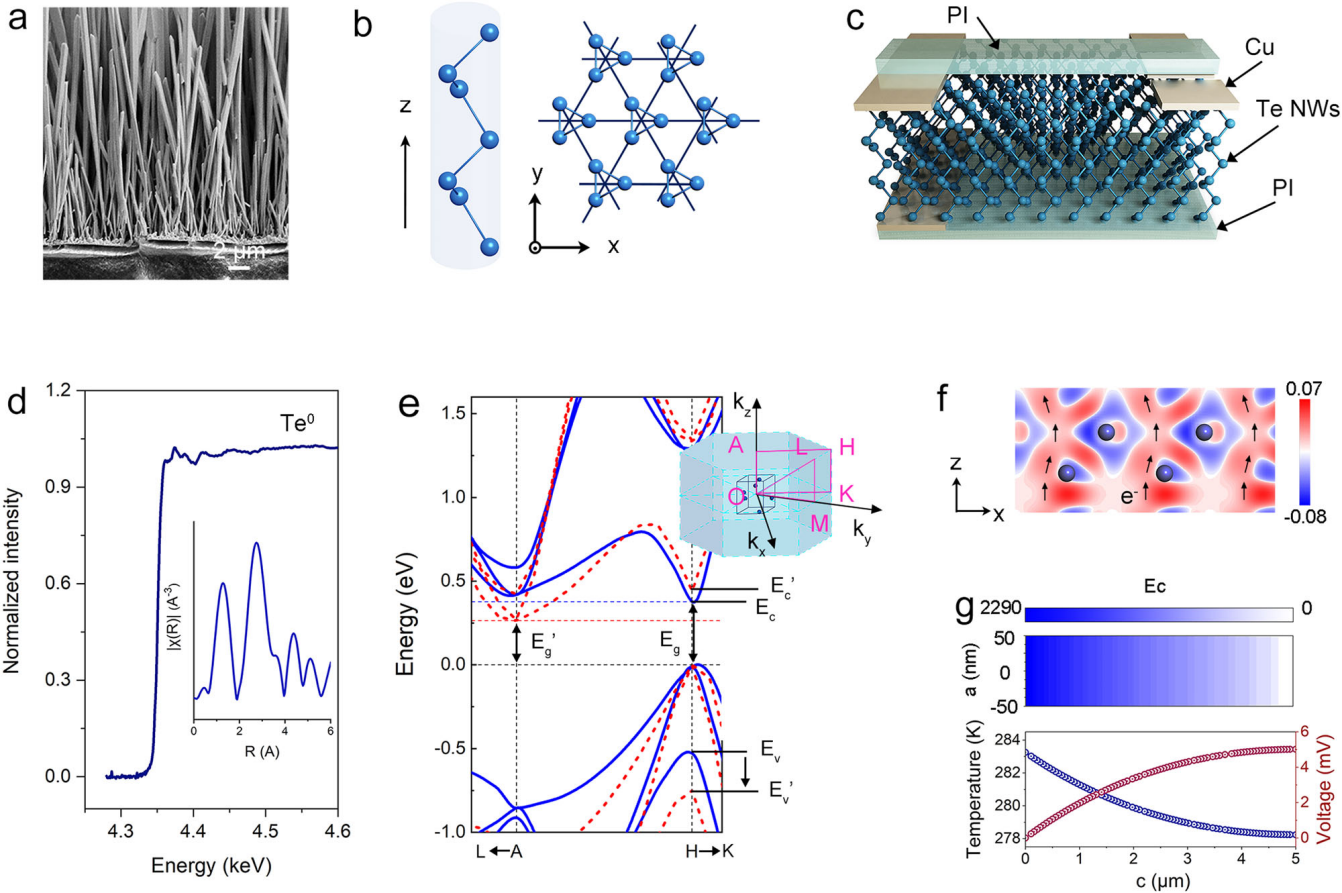
Structure and sensing characteristic of Te. **a** The side-view SEM image of Te NWs@PI. **b** Illustration of Te crystal structure. **c** Schematic diagram of DTS. **d** the X-ray Absorption Fine Structure (XAFS) of Te. **e** Band structure of Te. **f** Differential charge density diagram of Te crystal before and after applying 100 KPa pressure along the z axis. **g** When the Te NW with a temperature of 283.15 K at one end is in the air of 273.15 K, the temperature, electric potential and z-axis components of electric field intensity of the Te NW along the Z-axis.

Te is a typical semiconductor whose single-crystal nanostructure ensures that it can be easily prepared as nanosheets^25^, nanobelts^26^, nanorods^27^, etc. Vertical Te NW arrays were fabricated using a simple vapor transfer deposition (VTD) method (Fig. 1a), which has been reported in a few previous studies. Characterization analyses, including XRD and TEM, were performed to confirm that the Te NWs were single-crystalline. Supplementary Fig. 1 shows the XRD patterns of the Te NW arrays. The XRD peaks of the Te NW arrays show good agreement with those of PDF#36-1452, confirming the successful preparation of the Te NW array (Supplementary Note 1). A series of periodically perfect circular diffraction spots were observed in the selected area electron diffraction (SAED) patterns, indicating that the Te NWs are partially perfect single crystals (Supplementary Fig. 2b). It can be concluded from the HRTEM images of different parts that the Te NW is single-crystalline (Supplementary Fig. 2c and d).

The relative ease of preparation of the single-crystal Te NW is largely because of its crystal structure (Fig. 1b). A single-crystal Te semiconductor is composed of ternary helical chains of Te atoms, and the helical chains are bound to a hexagonal system by van der Waals forces. In this structure, the relatively heavy atomic mass and large phonon scattering between molecular chains result in a low lattice thermal conductivity. Te has a high electron transport rate and electrical conductivity within the molecular chain^28,29^. Therefore, it exhibits good thermoelectric properties and electrical conductivity along the z-axis, which makes it attractive as the active layer material for BTS.

Te-enabled sensing performance offers an excellent choice for OTM mode BTS on the one hand, whereas on the other hand, input signal decoupling is very difficult. To decouple the input signal, we designed the BTS as shown in Fig. 1c to measure the stress and temperature difference. The BTS was successfully fabricated, as shown in Supplementary Fig. 3. A vertical Te NW array was formed between the electrode and the polyimide (PI) substrate (Fig. 1a & Supplementary Fig. 4), half of which was gold-plated. Moreover, an SU-8 layer was spanned on the NW arrays to prevent short circuits from isolating the top and bottom Te layers and to protect the NW array, as shown in Supplementary Fig. 5. Finally, Cu was used as the top electrode on the thin PI film to finish the fabrication of the wearable photodetector textile (Supplementary Fig. 3). Details of the BTS fabrication method are described in the Methods section. The XAFS of Te stored at room temperature for half a year shows that its valence state is 0 (Fig. 1d)^30^, which demonstrates its high stability and suitability for BTS applications.

### Piezoresistive effects in BTS

When Te is exposed to pressure stimuli, the crystal deformation caused by pressure results in a change in the band structure^31^. To confirm this phenomenon, we performed first principles calculations using the Cambridge Sequential Total Energy Package (CASTEP) based on density functional theory (DFT) (Fig. 1e and Supplementary Fig. 6). It can be observed from the results in Fig. 1e that there are significant differences in the band structure of Te at 0 kPa (blue solid line) and 0.1 MPa (red dotted line) external pressures along <001 >. Taking point H in k space as an example, the energy band is pulled apart under pressure along the z-axis, the locality of the electron is weakened, and the expansibility of the atomic orbital is enhanced, as can be observed from the density of states (Supplementary Fig. 6). The number of high-energy states of the electrons increases indicating that electron transfer between atoms requires less energy and is significantly easier than that without applying pressure stimuli. The differential charge density diagram clearly demonstrates that the charge density around the Te atoms is significantly reduced and the charge between the Te atoms in the chain is significantly increased (Fig. 1f). The electrons are less constrained by atoms and can be transferred more easily between atoms^2^. The change in the energy band structure of path H to K shows that after the energy band has broadened, the energy band at point H becomes steeper, the effective carrier mass is reduced, and the carrier mobility increases, thereby decreasing the resistance. Although studies have shown that pressure can produce an electric potential, the orientation design of the NW array causes the internal electronic polarizations to cancel each other out, which also breaks the connection between the stress and the potential^32^. Results of theoretical calculations and those reported in previous studies show that pressure only changes the resistance of the device.

To design other functionalities of the device, the piezoresistive performance of the device should be carefully evaluated. Supplementary Fig. 7a shows the current as a function of pressure in the range of 0–5 kPa under a 100 mV bias voltage. As the external pressure increases from 0 Pa to 5 kPa, the current increases from the initial 2.82 μA to 141.82 μA. Furthermore, the internal conductance of the sensor is 2.82 × 10^−5^ S and its resistance is approximately 35.46 kΩ when the sensor is not under external pressure. the minimum external load applied to the sensor is 10 Pa, at which point the conductance is 1 × 10^−4^S and the resistance is 10 kΩ, as shown in Supplementary Fig. 7a. The current and external pressure function was fitted using I/μA = 75.3 × (P/kPa)^0.4^. In addition, the output current of the sensor is in the order of μA at a bias voltage of 0.1 V, which facilitates the measurement of subsequent data and reduces the need for subsequent circuits.

The response time is an important parameter for the sensor and determines, to some extent, the range and environment where the sensor can be applied. Supplementary Fig. 7b shows the piezoresistive response time of the BTS. The sensor exhibited an excellent response time to pressure of <5 ms and relaxation time of <10 ms. Because of the limitation of the electrochemical workstation, the applied bias voltage was changed to 1 V to improve the sampling frequency during the response time test, which highlights the advantage of a larger output current device. The sensitivity and response time of the sensor can meet the requirements for bimodal tactile perception. Moreover, the bimodal sensor exhibited good stability, as shown in Supplementary Fig. 7c. The response current remained roughly the same over 10,000 cycles at 2 Hz.

### Thermoelectric effect in BTS

When the sensor is exposed to temperature stimuli, the temperature difference between the device upper and lower surfaces of the device causes unilateral accumulation of carriers. The low-temperature side has a spatially high potential due to the accumulation of most carrier holes. In contrast, regions of high temperatures are characterized by low potential. This phenomenon is known as the Peltier effect^33^. A more direct representation of this phenomenon in Te NWs can be implemented using COMSOL Multiphysics simulations. When a heat source of 283.15 K is applied to the bottom of the Te NWs in the presence of air at 273.15 K, the temperature, voltage, and electric field in the Te NW vary with the position, as shown in Fig. 1g. When a temperature difference exists between the two ends of the Te NW, a temperature difference potential is produced at both ends owing to the Peltier effect. Existing studies have shown that within a small temperature range, the conductance changes minimally with temperature and can be neglected^34^. Therefore, a small temperature change from 20 °C to 80 °C only affects the output voltage of the BTS.

The thermoelectric properties of the BTS were further investigated. We first measured the temperature gradient-dependent (Δ*T*) thermoelectric voltage generation of a single sensor to evaluate the temperature-sensing response of the fabricated device. Considering the difficulty of temperature control and the fact that the expected application is a one-sided stable heat source, we used Peltier heating at the bottom of the device, and the upper part of the device to contact non-flowing air and generate a stable heat field. During the actual measurement, the temperature of the heat source at the bottom of the device was controlled using the applied voltage of the Peltier, and the actual temperature on both sides of the device was measured using a thermocouple thermometer. The laboratory temperature was maintained constant at 23 °C, and the measured output voltage was linearly dependent on the bottom temperature, with a coefficient of 0.317 mV/K (Supplementary Fig. 8a). Furthermore, the output voltage was approximately 3.6 mV at a bottom temperature of 36 °C, which is close to the surface temperature of the human body, indicating that the device can be used for human body-based waste heat collection or temperature detection. Supplementary Fig. 8b shows the dependence of the measured output voltage on temperature difference from 0 K to 17 K. There is a clear linear correlation between the output voltage and temperature difference, and the Seebeck coefficient is approximately 0.941 mV K^−1^, which is significantly higher than that of a Te block^35^.

To accurately reflect the response time of the device, we heated the bottom part of the device and waited for a stable temperature field to be established between the heat source, device, and air. When the temperature field is in equilibrium, a small temperature difference exists between the upper and lower parts of the device, which produces a parasitic potential; this is the reason the initial voltage in Supplementary Fig. 8c is not zero. Bulk aluminum (Al) stored at room temperature was placed on the upper surface of the device, and the variation curve of the output voltage with time is shown in Supplementary Fig. 8c. The measured temperature response time is <0.75 s and the relaxation time is approximately 5 s. The Al block was placed on the upper surface of the device; then, the particles were opened and heated. Supplementary Fig. 8d depicts the measured bottom temperature and output voltage as a function of time. The consistency of the two functions shows a good synchronization rate, which confirms the linear correlation between temperature difference and the output voltage of the device, indicating that the response time meets the requirements.

The above tests show that BTS exhibits excellent thermoelectric and piezoresistive performance, which provides the basis for the realization of perception. To realize bimodal perception in a single element, the two signal sources should not interfere with each other, and the coherence between them should be small, which is important for reducing decoupling difficulty and realizing bimodal sensing function. While previous separate tests showed that the interference between the thermoelectric and piezoresistive signal sources is negligible, additional tests intuitively showed that the coherence between them is small (Supplementary Fig. 9). In the first stage, there is no load at room temperature, and the voltage and current are the initial values. In the second stage, the bottom was heated to 40 °C, the voltage was increased and the current was constant. The first two stages show that the temperature difference and temperature have minimal effect on the resistance of the sensor. In the third stage, a room temperature (RT) Al block was placed on the sensor, and both current and voltage increased. The gravity of the Al block exerts pressure on the sensor, which increases the current. Furthermore, the thermal conductivity of the Al block is higher than that of air, which increases the temperature difference between the upper and lower surfaces. As a result, the output voltage increases. In the fourth stage, a weight was placed on the Al block. Heat dissipation on the upper surface of the device remained unchanged, and there is no obvious change in the output potential. However, when the mass of the external object increased, the current increases further. The latter two stages show that external pressure has no effect on the output potential of the device. In general, thermoelectric and piezoresistive effects do not affect each other.

### Mechanism of object classification

**Fig. 2:**
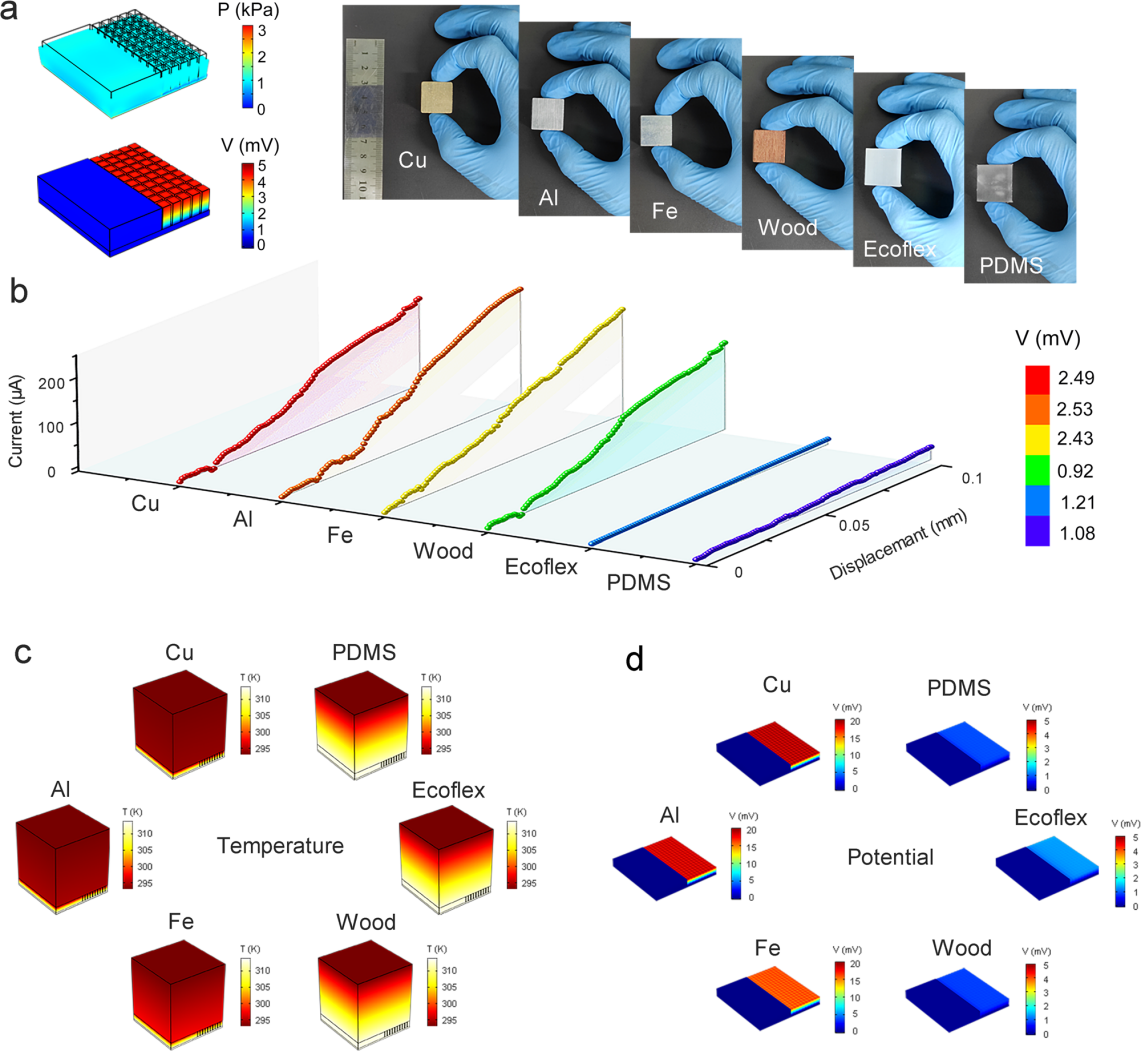
Mechanism of object classification. **a** The tactile sensor Internal stress distribution at 1 kPa (top of the picture), and potential distribution at the bottom of 313.15 K and room temperature of 293.15 K (bottom of the picture). **b** The relation curve between the current and the compression displacement of different object at 0.1 V bias voltage. The bottom heat source is 313.15 K, and the initial current is 20 μA under preloading. **c** The temperature distribution and **d** the potential distribution in the device are simulated when the device touches different objects.

The above results show that the device has an excellent detection rate, response speed, and robustness in temperature sensing and pressure sensing application. Moreover, the two signals did not interfere with each other. These characteristics provide a reliable basis for bionic bimodal tactile devices. Elastic modulus and thermal conductivity are two important basic parameters of materials, and they are also important labels to distinguish materials. At room temperature, the elastic modulus reflects the perception of the human skin as the softness of the object, whereas the thermal conductivity reflects the temperature of the object, which is the skin surface temperature. This conclusion will be theoretically validated through simulation calculations, as presented in subsequent sections (Fig. 2a). Inspired by the human skin, a dual-mode tactile sensing device was fabricated to simultaneously perceive the softness of materials and surface temperature and realize the partial recognition function of the skin.

For materials within the range of elastic strain, the relationship between stress and strain can be expressed as:

1 $$\sigma={{{{{\rm{E}}}}}}\varepsilon$$

Where *σ* is the biased pressure, *E* is the elastic modulus, and *ε* is the strain. A linear relationship can be clearly observed in Fig. 2b. The biased stress is proportional to the elastic modulus when the compression strain is constant^36^. The magnitude of stress can be expressed in terms of the device conductance; that is, the magnitude of the elastic modulus can be expressed using the output current of the device at a specific bias voltage. Under a uniform positive pressure, the distribution of the internal stress in the device is relatively uniform (top of Fig. 2a). Consequently, the internal conductance of the device increases uniformly and synchronously, indicating that there is no large local resistance inside the device due to structural problems that can result in poor device performance. In summary, the fabricated device can be used to distinguish between the softness and hardness of materials (Supplementary Table 1). In terms of thermal conductivity, a temperature difference exists between the fixed temperature at the bottom of the sensor and the top surface temperature, which can be expressed using the output voltage of the sensor (Fig. 2a).

To verify the mechanism of distinguishing material properties, we used BTS to detect the following six blocks of the same size and different materials: Ecoflex, PDMS, wood, Al, Fe, and Cu (Fig. 2b). The blocks were pressed down on the device at a constant speed of 0.01 mm s^−1^ at room temperature (23 °C). When an output current of 20 μA is considered the initial state, the curve of current relative to time is shown in Supplementary Fig. 10. The current can be expressed as a function of the displacement, taking the loading rate into account (Fig. 2b). When the compression displacement reaches 0.1 mm, the corresponding current of the metal and wood block is more than 200 μA. In contrast, rubber blocks yielded a current value of only 50 µA. Metal and wood blocks are much harder than rubber blocks and exert more pressure under the same compression displacement, which results in a higher current. Therefore, bimodal sensors can sense the hardness of a material.

After verifying the hardness characterization of the blocks, the next step is to characterize the thermal conductivity of the blocks using the bimodal tactile sensor. A square block was placed on the upper surface of the sensor, and a heat source of the same temperature and power was placed on the lower surface of the sensor. The V-t curve of the bimodal tactile sensor is shown in Supplementary Fig. 10. When the temperature field reaches approximately dynamic equilibrium after 1 min, the thermoelectric potential corresponding to PDMS, Ecoflex and wood blocks is approximately 1.0 mV, whereas the thermoelectric potential corresponding to metal blocks can reach approximately 2.5 mV (Supplementary Fig. 10). In Fig. 2b, the colors of the lines correspond to the different thermal voltages that were measured when the system is in thermal equilibrium; the figure shows that the thermal potential of metals is much higher than those of other materials. At the material level, the thermal conductivities of rubber and wood are much lower than that of metal. This results in energy accumulation in the contact area between the rubber and the upper surface of the device, leading to an increase in the upper surface temperature, a decrease in the temperature difference between the two sides of the device, and a corresponding decrease in the output potential. Evidently, the device can be used to identify the thermal conductivity of materials.

Furthermore, we used COMSOL Multiphysics to verify different thermal conductances corresponding to different thermal voltages. When the upper surface of the device is in contact with the metal, heat is transferred to the metal along the temperature gradient. Because of its good thermal conductivity, heat can be rapidly diffused to the interior and will not accumulate near the contact surface. The temperature distribution near the metal block and the contact surface was relatively uniform and low (three illustrations on the left of Fig. 2c). For the other three materials with lower thermal conductivities, a greater barrier to heat transfer along the temperature gradient exists, making it more difficult for heat to diffuse through the block, resulting in heat accumulation near the contact surface. The temperature difference is mainly distributed inside the block, whereas the temperature difference between the two sides of the device is small (three illustrations on the right of Fig. 2c). Figure 2c shows the difference in temperature distribution caused by different thermal conductances based on the heat diagram. The distribution of the potential produced by different thermal conductances can be easily obtained using the formula ${V}_{{therm}}={{{{{{\rm{S}}}}}}}_{T}\,\times \,\triangle T$ (Fig. 2d). A higher thermal conductivity corresponds to a higher potential, as shown in Fig. 2d. Further, the results confirmed the mechanism by which the sensor acquires the thermal conductivity information of the material.

### Decoupling of resistance and voltage signals

The above result verifies that the change in resistance can reflect the hardness of the contact material, and the thermal conductivity of the material can be measured using the electric field within the material. However, there are still two problems associated with BTS that need to be solved urgently. First, the displacement parameters need to be controlled using a mechanical platform, which significantly limits the application areas of BTS. Second, the measurement circuits for voltage and resistance are significantly different. Simultaneous detection of voltage and resistance in a single element increases the complexity of the other circuits and introduces interference from the instrument.

**Fig. 3:**
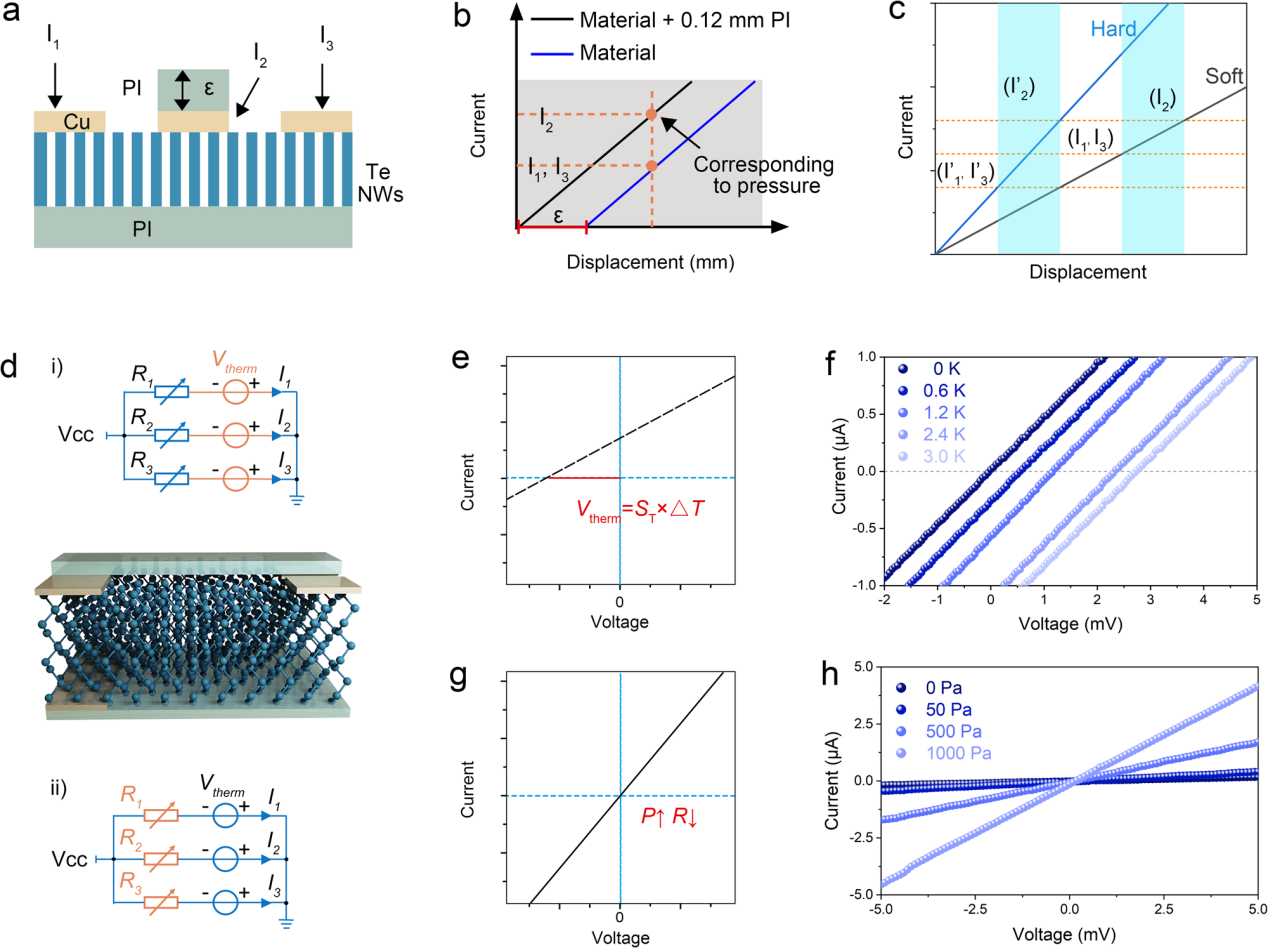
Signal preprocessing of BTS. **a** Cross section structure of the DTS. **b** Ideal curves of current and displacement in different channels. **c** Ideal curves of current and displacement for materials to be identified with different hardness. **d** Decoupling mechanism of two signals in DTS. The ideal IV curve for both stimulus signals: **e** temperature difference and **g** stress. **f** The IV curves of DTS under different temperature differences. **h** The IV curves of DTS under different stress.

A fixed compression displacement *ε* was introduced to eliminate the dependence on the displacement parameter, as shown in Fig. 3a^37^. Although there is a more complicated functional relationship between resistance and strain, as well as strain and displacement, it can be observed from Fig. 2b that the relationship between current (reciprocal of resistance) and displacement is approximately linear. Therefore, in an ideal state, when the displacement is zero, the middle part of the BTS first touches the object. When the contact object compresses and drops by 0.12 mm, both sides of the BTS begin to make contact with the object. The corresponding change in the output current of the device is shown in Fig. 3b. It can be observed from the figure that I_2_ directly reflects the force used in contact, whereas the difference between I_2_, I_1_, and I_3_ is related to the hardness of the contact object. Contact objects with different degrees of softness and hardness correspond to different current strain curves, as shown in Fig. 3c. When the force used for touching is the same, the output currents of the devices on both sides of the BTS are significantly different. Hardness can be expressed by the correlation function of I_1_, I_2_, and I_3_. This is simplified as $\varepsilon=F\left({I}_{2},\frac{{I}_{1}+{I}_{3}}{2{I}_{2}}\right)$, considering the symmetrical structure of the device, indicating that the hardness of the contact object can be derived independent of displacement.

Another problem is the simultaneous measurement of current and voltage in a common circuit. The BTS was simplified as a variable resistor and voltage source (Fig. 3d). The current on the branch can be expressed as:

2 $${I}_{i}=\frac{1}{{R}_{i}}\left({V}_{{cc}}-{V}_{{therm}}\right){{{{{\rm{;}}}}}}\,i=1,2,3$$

From the test results, the BTS resistance does not change with the external voltage, which also demonstrates that a good ohmic contact is formed at both ends of the device (Supplementary Fig. 11). Interestingly, two points can be used to determine the curves of *I*_*i*_ and *V*_*cc*_. In a linear IV curve, the intercept of the horizontal axis represents the *V*_*therm*_ of the BTS (Fig. 3e), and the resistance of the BTS can be represented by the reciprocal of the derivative of the curve (Fig. 3g). In other words, the resistance and voltage of the BTS can be obtained by measuring only the branch currents in the two *V*_*cc*_ states (Supplementary Note 2 and Supplementary Note 3). In this way, we could synthesize a true OTM sensing unit, which is neither a superposition of a single OTO sensor nor a separate characterization of two incompatible electrical parameters (Supplementary Table 2 and Supplementary Note 4). When the device is subjected to temperature stimulation, the slope corresponding to conductivity remains the same as the resistance, and the transverse intercept corresponds to *V*_*therm*_, which varies linearly with variations in Δ*T* (Fig. 3e). Figure 3f illustrates that the IV curve of the device shifted significantly, demonstrating that the device outputs a corresponding *V*_*therm*_ as the temperature difference changes. The slope remained constant, indicating that the resistance is not sensitive to temperature. Figure 3g shows the theoretical IV curve of a typical piezoresistive device, where the slope of the curve corresponding to conductivity maintains the same trend under stress. Figure 3h shows the experimentally measured IV curve of the BTS. The slope of the curve, that is, the resistance, changes significantly with an increase in pressure. However, the transverse intercept of the curve does not change significantly, meaning that no obvious electric potential is generated at the scale of 5 mV, which is close to the range of the thermoelectric potential. These results show that the voltage is not sensitive to stress. The above section shows that the BTS could realize the simultaneous detection of two signals in the OTM mode.

### Tactile perception system based on BTS

**Fig. 4:**
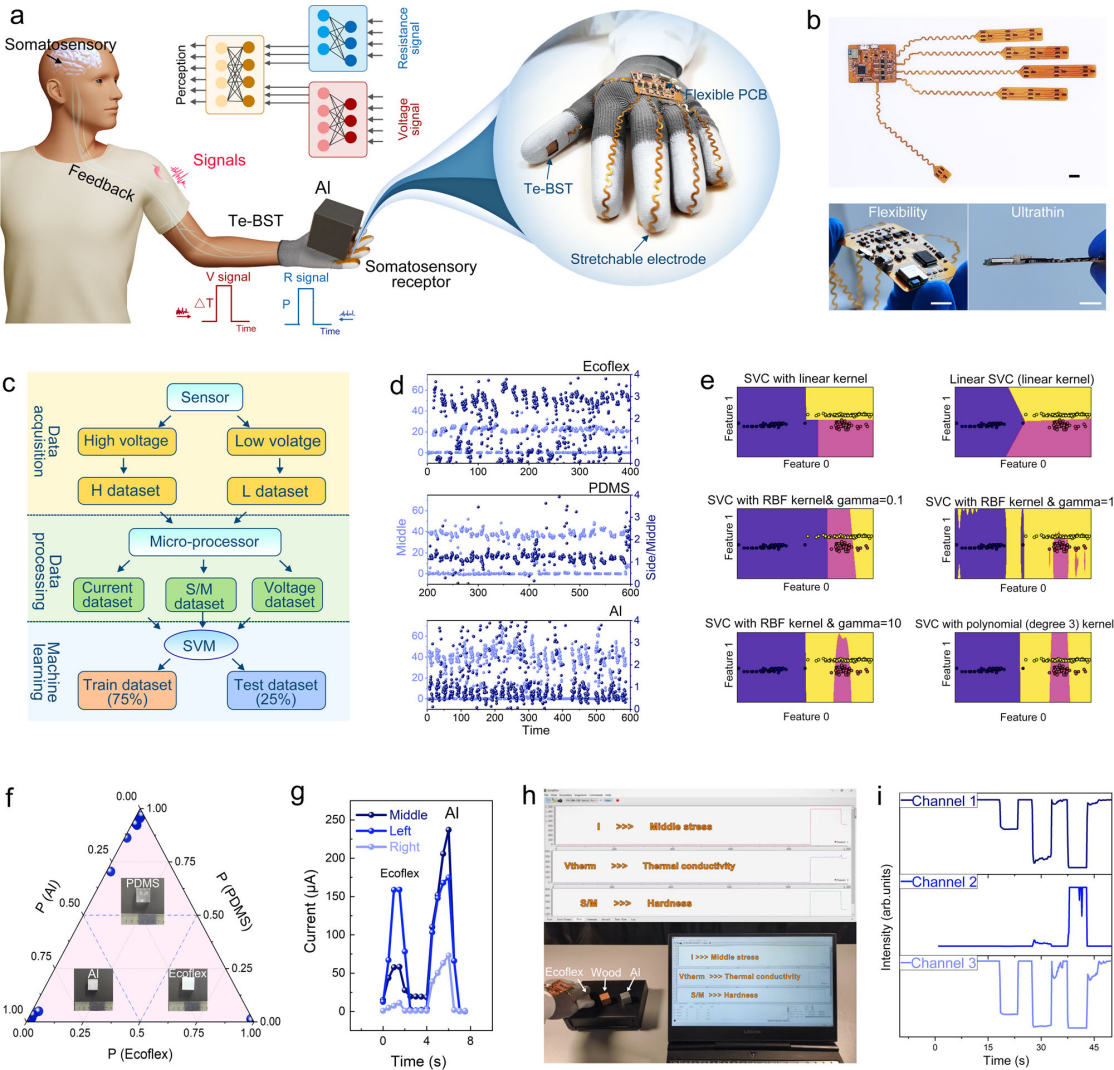
A bionic tactile perceptive system based on BTS. **a** Schematic diagram of tactile perception working. The illustration depicts the BTS-based smart glove. **b** The photograph of sensor and processor in bionic tactile perceptive system based on BTS. Scale bar: 1 cm. **c** Flow chart of machine learning for material recognition. **d** The current dataset and S/M dataset of Ecoflex, PDMS and Al under repeated pressing. **e** The train dataset is classified via support vector machine (SVM) with different kernel functions. **f** The probability of test dataset classification is calculated by using the SVM model after training. **g** Unprocessed H dataset of Ecoflex and Al. **h** The process of identifying different materials. **i** The corresponding dataset in h.

The human skin is an important organ used by the body to perceive external information^38,39^. We proposed a BTS-based smart glove that mimics human skin and possesses the basic cognitive abilities to perceive external stress and temperature (Fig. 4a). Stress and temperature differences produce changes in resistance and voltage, respectively. The initial processed electrical signals are accepted by nerves using the increasingly developed man machine interface^40–42^ and transmitted to the somatosensory system, which processes this electrical signal and provides feedback based on the cognitive results. As a demonstration application of this glove, we built a tactile perception system that can help people identify materials. The system collects material property information through tactile sensors attached to five fingers (top of Fig. 4b), which is processed into three feature values using a microprocessor and displayed in a user interface in real time. In this system, the processor performs the role of the somatosensory system to carry out data processing and classification. This system is made into a flexible, ultra-thin printed circuit that can be properly deformed by hand movements (bottom of Fig. 4b). Figure 4c depicts a schematic diagram of the material identification. The current data obtained from the sensor at a high *V*_*cc*_ voltage of 100 mV is classified as the H dataset (Fig. 4g), and the current data obtained when the sensor operates at a low voltage of 0 V is classified as the L dataset. The H and L datasets were processed using a microprocessor into three sets of data sequences: current, S/M, and voltage datasets.

The current dataset reflects the pressure applied by the finger; the S/M dataset reflects the hardness of the material; and the voltage dataset reflects the thermal conductivity of the material (Fig. 4d and Supplementary Fig. 12). In Fig. 4d, the three groups of middle current were similar and fluctuated slightly in the same material recognition, respectively reflecting the habitual force during touch and the inaccurate control of the mechanics by the human body. It further proved that the self-locking structure in a single device is indispensable for hardness testing. The three sets of data were summarized into a sample set of 240 samples, and we used the support vector classification (SVC) algorithm with the different kernel function in the support vector machine (SVM) for machine learning. We used 75% of the sample for training to obtain a suitable classification model (Fig. 4e). It can be observed from the comparison of several types in Fig. 4e that the linear kernel is now suitable and no further dimensionality reduction processing is required. The sample points are clearly distinguished in two dimensions, namely, thermal conductivity and hardness, but there is an overlap in a single dimension, making it difficult to distinguish (Supplementary Fig. 13a, b). This highlights the advantages of multi-mode sensors over single-mode sensors^17^. The remaining 25% of the sample was used for classification testing via the modal, and the classification probabilities of the test sample points are shown in Fig. 4f. It can be observed from the Fig. 4f that the samples are distributed in three vertex angles and the distribution in the central region is zero, which indicates that the sample with fuzzy classification is zero. Depending on the stable performance of BTS, a very high determination accuracy was achieved with less training.

The tactile perception system attached to a human hand was successfully applied to classification demonstration (Fig. 4h). Three data series of three materials were obtained through the smart gloves, and there were obvious differences between them (Fig. 4i and Supplementary Movie 1). When the softest Ecoflex is touched, the deformation is rather considerable, and more pressure is shared on both sides, resulting in output of channel 1 being significantly lower than that of the other two groups. The high thermal conductivity of aluminum, on the other hand, makes value of channel two substantially greater than that of the other two groups (Supplementary Fig. 14). A linear kernel was used for classification, and the resulting criterion was used to identify the material. The successful application of BTS at the fingertips also makes it easy to transplant to machinery with precise displacement control (Supplementary Fig. 15 and Supplementary Movie 2).

### Interaction with the VR space

The rise of the meta-verse is inseparable from the development of related technologies such as VR^43^. However, the majority of current research, is confined to using body motions to control the VR world directly, with no link to objects in the real world (Supplementary Table 3). We wish to transfer certain genuine items in the physical world to the VR space, improve communication between the VR and real worlds, add some building pathways to the virtual area, and expand the VR interactive capabilities. Here, the BTS-based smart glove was tried for an interaction with the VR space.

**Fig. 5:**
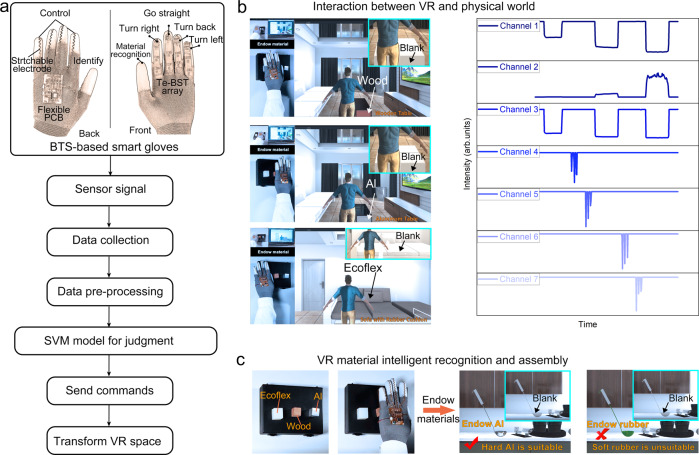
The demonstration of VR interaction using the BTS-based smart glove. **a** The flow chart for material recognition and interaction. **b** Interaction between VR and physical world. Use the surrounding materials to modify the furniture in the VR world according to your wishes. Right of the graph illustrates seven data sequences for seven dismantling actions, which included giving Ecoflex, right turn, forward, giving wood, backward, left turn and giving Al. **c** Assemble the spoon by touching the correct material according to the material properties required for the spoon.

As the flow chart shows in Fig. 5a, the current signal from glove was collected by the processor and preprocessed into seven data series (right of Fig. 5b). The seven datasets were judged by the model obtained by SVM training. Then, the processor sent corresponding commands to Unity through serial communication. The VR space was transformed according to the corresponding commands. In the seven data series, the data of the first three channels were derived from the BTS on thumb and used for material recognition. In addition to the material recognition, movement in the VR world is also essential. Therefore, only taken was the current dataset part of BTS output sequence on the remaining four fingers, and its function is simplified as a pressure sensor for movement control. Shown as Fig. 4a, b, channel 4 was the output dataset of BTS on the index finger, which was used to control the right turn; the BTS data outputs on the middle, ring and little finger correspond to channels 5 to 7 respectively, which control the forward, backward and left turn of the characters in VR space in turn (Supplementary Fig. 16 and Supplementary Movie 3). The right of Fig. 5b shown seven data sequences for seven dismantling actions, which included giving Ecoflex, right turn, forward, giving wood, backward, left turn and giving Al. In this way, the interaction in the VR space through the smart glove was relatively complete. This glove was demonstrated to be used to endow furniture in the VR world with material in physical world according to the wishes of wearer (Fig. 5b, left and Supplementary Movie 4). A table and a sofa were given textures according to the preference of wearer (Supplementary Figs. 17-18), and more importantly these textures were derived from objects around the wearer in the real world. This is an attempt transform the VR world according to the real world and a push to strengthen the connection between the VR world and reality. In addition, a puzzle game to assemble a spoon was shown as Fig. 5c (Supplementary Fig. 19). The spoon body need a material with a high hardness to maintain its shape, and the spoon handle need a small thermal conductivity to insulate the heat. Only by selecting the material of the attribute properties for each part, can a complete spoon be spelled out (Supplementary Movie 5).

### Neural reflex circuit based on BTS

**Fig. 6:**
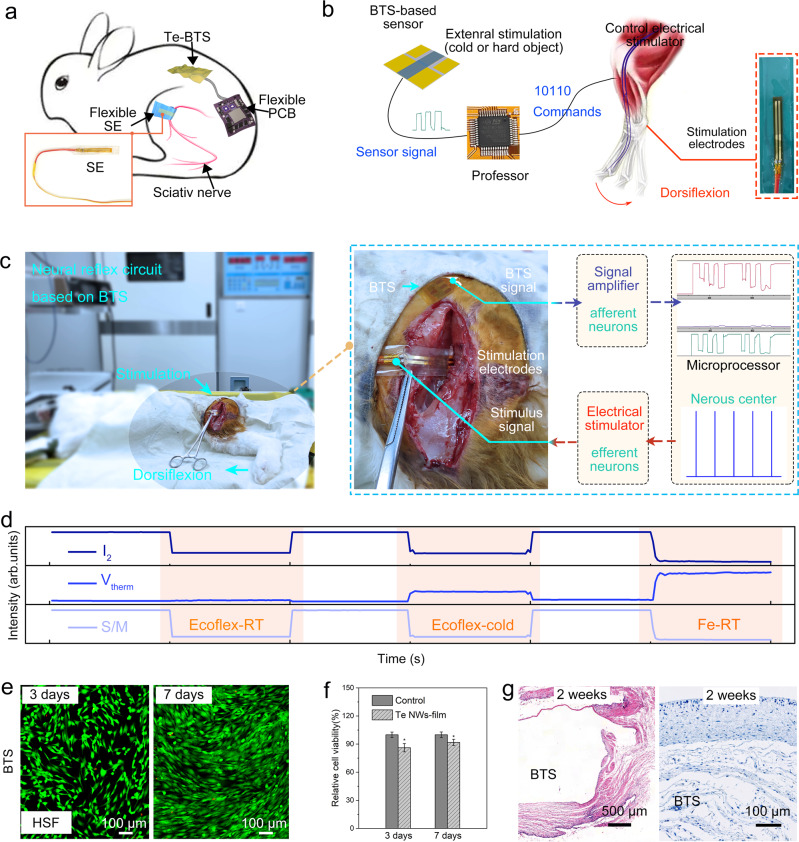
a Characterization of BTS biocompatibility. **a** Schematic of neural reflex circuit based BTS. **b** Schematic diagram of signal transmission in neural circuits. The spacing of the stimulation electrodes is 1 cm. **c** The rabbit regains its senses via the bionic tactile perceptive system. General anesthesia deprived the senses of rabbit. The system identifies the external stimulus and controls the impulse signals to stimulate the sciatic nerve based on the results, so as to realize the body reflex. **d** The external stimulus signal acquired by the system: Ecoflex at RT condition, RT Al, Ecoflex at cold condition. Results after three and seven days of cell culture on the device: **e** number of live/dead cells and **f** relative cell activity. Data are represented as mean±S.D. Statistical analysis was performed one-way analysis of variance (*n*=4 × 10^4^ cells/mL examined over 3 independent experiments, ^*^P < 0.05). **g** Results of paraffin sections for H&E staining (left) and frozen sections for immunohistochemistry (right). A fibrous capsule of comparable width is formed around both the BTS and silicone (control) samples at 2 weeks.

Figure 4a depicts the schematic diagram of the normal human reflex circuit. External stimulation generates nerve impulses, which are transmitted to the parietal area responsible for somatosensory function. After processing by the cerebral cortex, the control signal is output to control the movement of skeletal muscles. We designed a neural reflex circuit based on the BTS as a conceptual demonstration of a complete system of tactile perception and performed stress reaction to an external threatening stimulus (Fig. 6a–c).

In the system, the rabbit was anesthetized and unable to respond to external stimuli, which can be interpreted as loss of skin perception. The BTS was stitched onto the skin, allowing the rabbit to regain skin perception. The BTS generates a signal similar to a nerve impulse, which is directly transmitted to microprocessesor on FPCB. The microprocessor processes the stimulus signals, analyzes them and relays instructions to the executing unit via Bluetooth. The instruction was used to command a 1 Hz continuous pulse, which control the dorsiflexion of the rabbit by a stimulation electrode connected to the sciatic nerve. Shown as Fig. 6c, nerve impulses are afferent to the nerve through the afferent nerve, and stimuli are sent to the microprocessor via amplification of the circuit on the FPCB. The processing of the external stimulation signal by the central nervous system and the nerve signal transducer in the spine is similar to the processing of the external stimulation signal by the microprocessor and regulating the stimulator to create a 1 Hz stimulation signal. The instructions reach the skeletal muscle through the efferent neurons after a sequence of processing in the center, causing the skeletal muscle to contract. Three materials were selected as the stimulus sources for the experiment to mimic the cold and hard stimuli in a real world, namely Ecoflex blocks and iron blocks stored at RT and Ecoflex blocks refrigerated in a refrigerator, which could stimulate the sensor to produce three distinctive signals analyzed and determined by the processor. Because Ecoflex (RT) is temperature-friendly and very soft, there is no stress response to its stimulation. When the Ecoflex (cold) stimulus was applied, the processor determined that the cold stimulus was strong and threatening, and then controlled the stress response of the rabbit dorsiflexion. Similarly, when Fe stimulation was used, a hard object was detected, and the result was the same for the rabbit dorsiflexion (Supplementary Movie 6). The results show that tactile sensing based on dual-mode tactile sensors can theoretically help to regain tactile perception, and with further advancement in brain-computer interfaces and artificial nerves, endowing of tactile perception will gradually become a reality.

### Biocompatibility

Although the device is currently attached to the body surface, good biological compatibility is still of great significance. First, the mechanism of perception means long-term skin contact, and good biocompatibility is a safety guarantee for long-term replacement and even permanent use. In addition, good biocompatibility allows the device to become part of the body tissue. Therefore, biocompatibility and cytotoxicity tests of the devices were conducted. Human skin fibroblasts (HSFs) were cultured on the device as the experimental group (Supplementary Fig. 20), whereas HSF was cultured in a blank medium as the control group. The fluorescent images in Fig. 6e show the survival status of the cells after 7 days (168 h). In the live/dead assay, there were many live cells (green) and few dead cells (red), with minimal difference between the experimental and control groups. After 7 days (168 h), the relative cell viability on the sensor was similar to that in the control group and increased compared with the experimental group culture for 3 days (Fig. 6f). Two weeks after the device was implanted in a rabbit, a clear fibrous capsule was formed around the device, and there was no obvious inflammation around the tissue (Fig. 6g and Supplementary Fig. 21). These data indicate that the dual-mode sensor is non-toxic and has good biocompatibility^19,44^, which is important for biomedical applications.

## Discussion

We have reported a bright dual-parameter sensor based on the anisotropy of Te NWs that transduce different stimuli into separated signals to intrinsically minimize signal interference, allowing for sensitive detection of both temperature and pressure in a single device and realize VR interaction and neuro-reflex applications. A BTS-based smart glove was constructed based on this sensor, and thermal conductivity and hardness properties were learned and classified using a deep learning technique. Further, a unique interaction mechanism between VR and reality was built based on this glove, which gives an inspiration for the development of meta-verse. Finally, we designed a stimulus-feedback neural circuit that enables the body to respond to threatening stimuli. Experimental results demonstrate the portability of the artificial touch function and its prospects in biological medicine. Good biological compatibility also provides an excellent guarantee for application in biological medicine.

## Methods

### Pretreatment of substrate

The polyimide (PI) was cleaned with ethanol ultrasonic for 30 min, and then cleaned with deionized water. 10 nm Cr and 50 nm Au were successively evaporated on PI films by thermal evaporation.

### Synthesis of Te NW arrays

Take 1 cm^2^ of PI steamed with Cr/Au as the substrate. The substrate was stuck on the downstream temperature drop zone of tube furnace, and 50 mg was put in the heating zone. Then, continuously pass argon with a flow rate of 50 sccm (standard cubic centimeters per minute) while maintaining the pressure below 20 Pa. Switch on the PE source to generate plasma, and raise the temperature to 500 °C in 35 min and keep it for 30 min. When the temperature dropped to room temperature naturally, Te NWs could be obtained on the substrate.

### Device fabrication

SU-8 was applied on the Te NWs with a rotation of 700 rpm (Revolutions Per Minute) for 30 s. After curing the SU-8 layer at 90 °C, the upper electrode is extracted with copper tape. A small piece of PI film is then padded in the middle to create a height difference (Supplementary Fig. 2).

### Material characterization

The morphology of the NWs was observed by Scanning electron microscopy (SEM, SU8020). The lattice structure was characterized by an X-ray powder diffractometer (Rigaku D/Max-2550, λ = 1.5418 Å) and transmission electron microscopy (TEM, JEOL JEM-2010F). This part was processed at room temperature.

### Simulations

Ab initio calculations were performed utilizing Cambridge Sequential Total Energy Package (CASTEP) based on the density functional theory (DFT). the generalized gradient approximation (GGA) of Perdew-Burke-Ernzerhof (PBE) was adopted to handle The electron exchange-correlation functional. In geometric optimizations and self-consistent calculations, the energy convergence threshold was set to 10^−5^ eV and 230 eV was adopted as the kinetic energy cutoff of the plane wave. External pressure is applied along the Z axis of the Te crystal.

The device simulations were conducted with COMSOL Multiphysics. The common conductive layer at the bottom is 200 μm × 200 μm × 5 μm Au. The gold-plated side of the NW is simplified into a block set at 200 μm × 100 μm × 20 μm Te1. The NWs are set as 8 μm × 8 μm × 20 μm micro column Te2, and the upper layer is a 200 μm × 200 μm × 200 μm contact material. The specific material parameters are provided in Supplementary Table 4 (Supplementary Note 1). Set transient study, balance can be reached within 1 s (Supplementary Fig. 22).

### Device characterization and measurement

All tests were carried out at room temperature of 23 °C. The bottom heat source is provided by semiconductor ceramic refrigerating sheet (TEC-12706) and The temperature is read by a digital thermometer (UNI-T, UT325). The electrical performance of sensor was characterized by Keithley B1500A and CHI 760E. The instron-E1000 was utilized to control pressure on the device.

### Cell biocompatibility in vitro

Human skin fibroblasts (HSFs) (iCell Bioscience Inc, ShangHai, China) were inoculated on a special medium for HSFs (iCell Bioscience Inc, PriMed-iCell-003) at a density of 4 × 10^4^ cells per pore, and cultured in an incubator (Shinetek, WIGGENS WCI −180) at a constant temperature of 37 °C under 5% CO2. The HSFs were cultured in vitro for 72 (3 days) and 168 h (7 days), washed three times with phosphate-buffered saline (PBS, Procell, WH0112201 911XP). Half stained with 10 μL calcitonin-AM and 10 μL polyimide for 15–20 min, was used to observe the number of live/dead cells, and the other half incubated for another 4 h after adding 0.5 mg/mL MTT (Solarbio, M8180) was used to observe the relative cell activity.

### In vivo biocompatibility assessment

The biocompatibility of BTS and silicone (control) were evaluated histologically. The sterilized BTS was implanted under the skin on the side of the spine of the rabbit, and sterilized silicon was implanted in the corresponding position on the opposite side as a control group. After normal feeding for 2 weeks, the tissues surrounding BTS were harvested from rabbits euthanized by CO_2_. The samples were then divided longitudinally into two part; one part was made into paraffin sections for H&E staining and the other part was used as frozen sections for immunohistochemistry.

### Animal experiments

All animal experiments were conducted in accordance with the regulations of Beijing Laboratory Animal Management Office (approval number: MDSW-2021-053C). A four-month-old New Zealand rabbits was used in the experiment. The rabbit was anesthetized. Remove the hair from the leg and cut through the tissue to find the sciatic nerve. The device is sutured above the wound and electrodes are stimulated to attach to the sciatic nerve. After the experiment, the rabbit was euthanized.

### Statistics and reproducibility

The statistical analyses were performed using Origin. No data were excluded from the analyses. In the experiment of biological compatibility, the experimental group and the control group maintained the same conditions except for the introduction of BTS. And each group contained three parallel experimental groups. In addition, the device preparation process was repeated more than five times, and the device performance showed similar results. The repeatability of the experiment is guaranteed.

### Reporting summary

Further information on research design is available in the Nature Research Reporting Summary linked to this article.

## Supplementary information

Supplementary Information

Description of Additional Supplementary Files

Supplementary Movie 1

Supplementary Movie 2

Supplementary Movie 3

Supplementary Movie 4

Supplementary Movie 5

Supplementary Movie 6

Reporting Summary

## Data Availability

The authors declare that the data that support the graphs within this paper are available from the corresponding author upon reasonable request. Source data are provided with this paper.

## Source data

Source Data

## Code availability

The codes used for simulation and data plotting are available from the corresponding authors upon request and are also available in Zenodo (10.5281/zenodo.7010536).
