# Supplementary material for: Dual sensing signal decoupling based on tellurium anisotropy for VR interaction and neuro-reflex system application
Source: Nat Commun. 2022 Oct 10;13:5975. doi: 10.1038/s41467-022-33716-9 (PMC9550802; doi:10.1038/s41467-022-33716-9)
Supplement: Supplementary file 1 — Supplementary Information [file 41467_2022_33716_MOESM1_ESM.pdf]

Dual Sensing Signal Decoupling based on Tellurium Anisotropy for VR Interaction and Neuro-Reflex System Application

Linlin Li[1,2], Shufang Zhao[1,2], Wenhao Ran[1,2], Zhexin Li[1,2], Yongxu Yan[1,2], Bowen Zhong[1,2], Zheng Lou[1,2], Lili Wang[1,2]* and Guozhen Shen[3]*

1. State Key Laboratory for Superlattices and Microstructures, Institute of Semiconductors, Chinese Academy of Sciences, Beijing 100083, China.

2. Center of Materials Science and Optoelectronic Engineering, University of Chinese Academy of Sciences, Beijing 100083, China

3. School of Integrated Circuits and Electronics, Beijing Institute of Technology, Beijing 100081, China

Correspondence and requests for materials should be addressed to L. L. Wang (liliwang@semi.ac.cn) and G.Z. Shen (gzshen@bit.edu.cn).

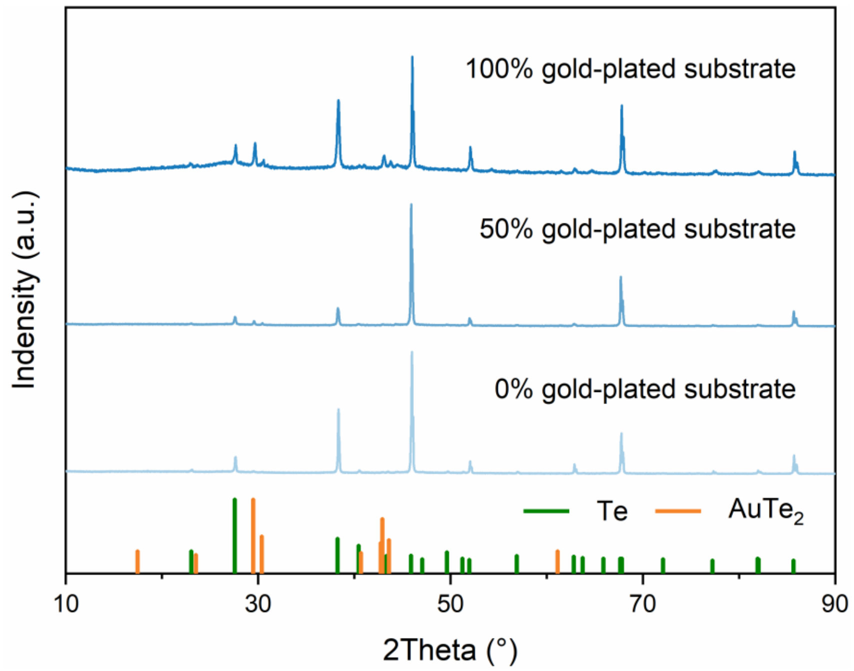

Supplementary Fig. 1 The X-ray diffraction (XRD) pattern characterization. The XRD pattern of Te NW array prepared on substrates with different gold-plated area ratios: 0%, 50% and 100%. XRD card for reference, Te: PDF#36-1452, $AuTe_2$: PDF#43-1472.

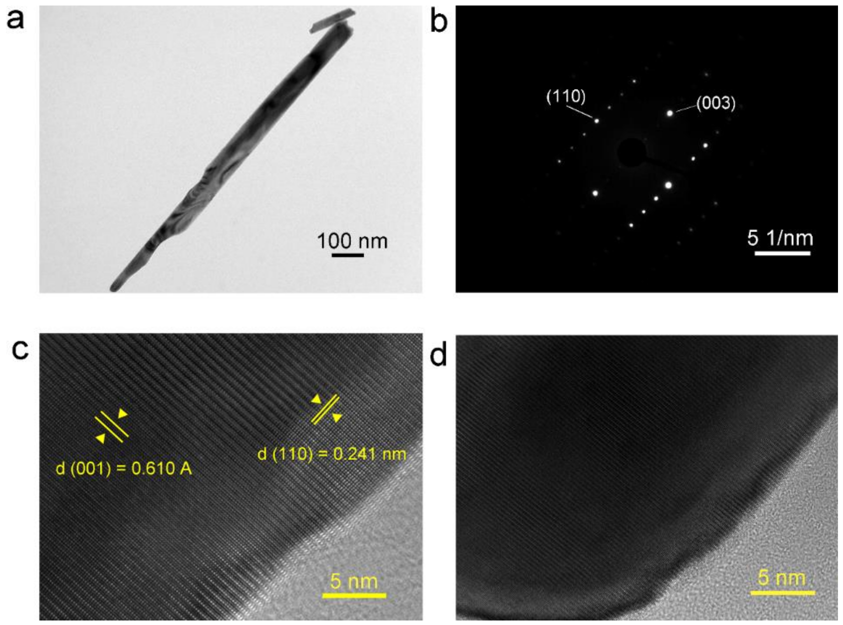

Supplementary Fig. 2 The TEM characterization of Te NW. a The TEM image of Te NW. **b** Selected area electron diffraction (SAED) patterns. **c** HRTEM image of the middle part. **d** HRTEM image of the tip.

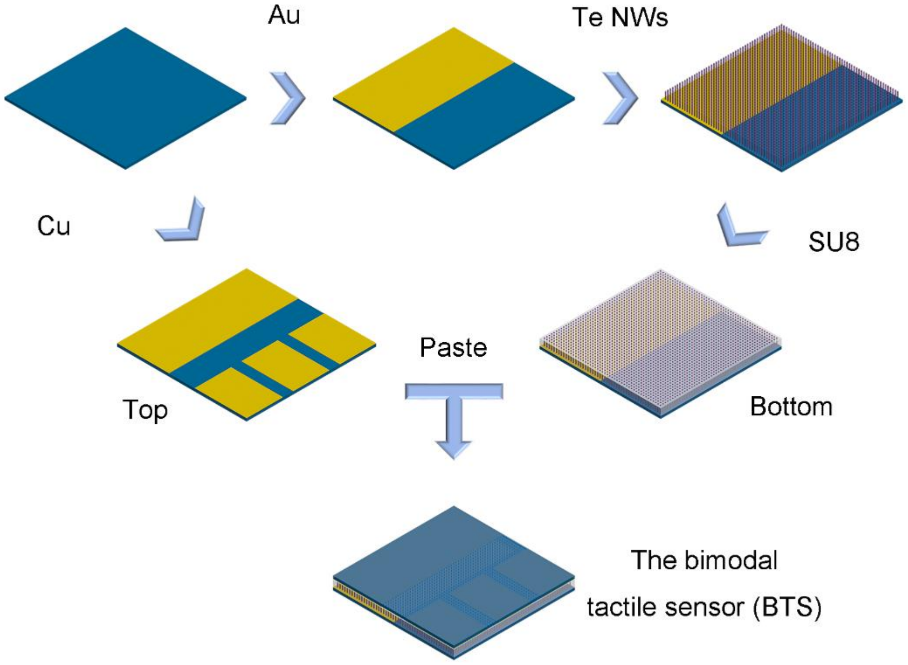

Supplementary Fig. 3 The flow chart of the sensor fabrication. Illustration of fabrication of the BTS via VTD method.

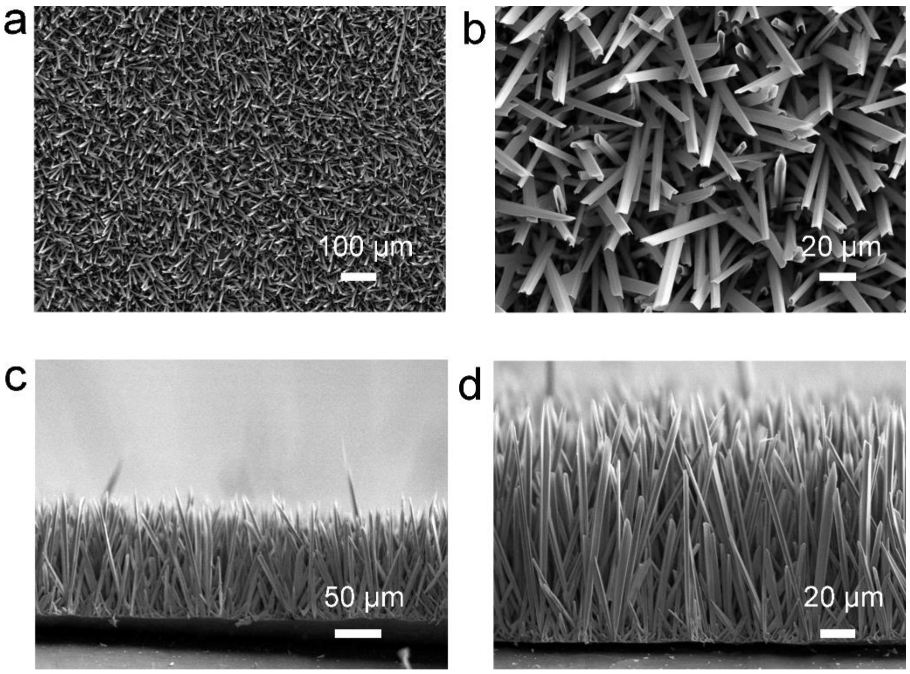

Supplementary Fig. 4 The SEM characterization of Te NW array. a Top view at low-magnification. **b** Top view at high-magnification. **c** Cross section at low-magnification. **d** Cross section at high-magnification.

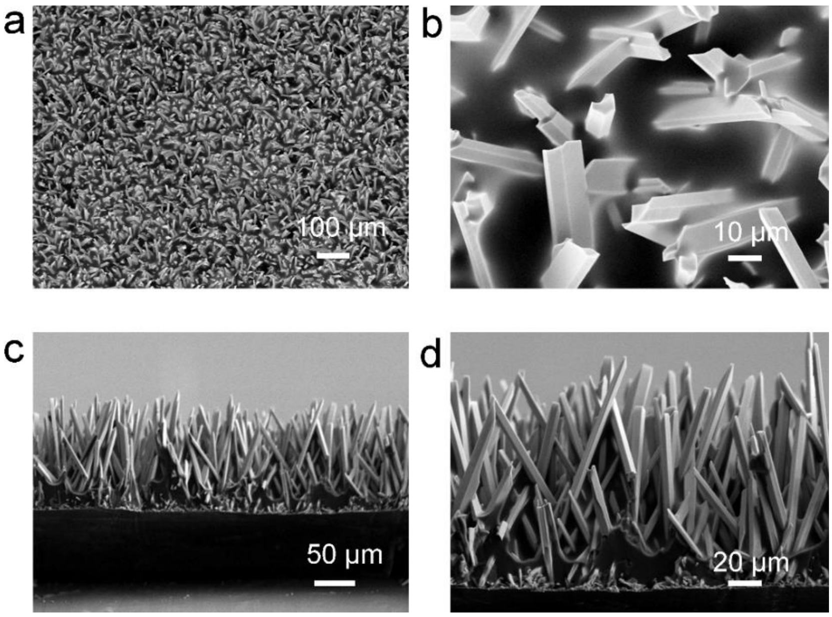

Supplementary Fig. 5 The SEM characterization of Te NW array assembled with SU-8. a Top view at low-magnification. **b** Top view at high-magnification. **c** Cross section at low-magnification. **d** Cross section at high-magnification.

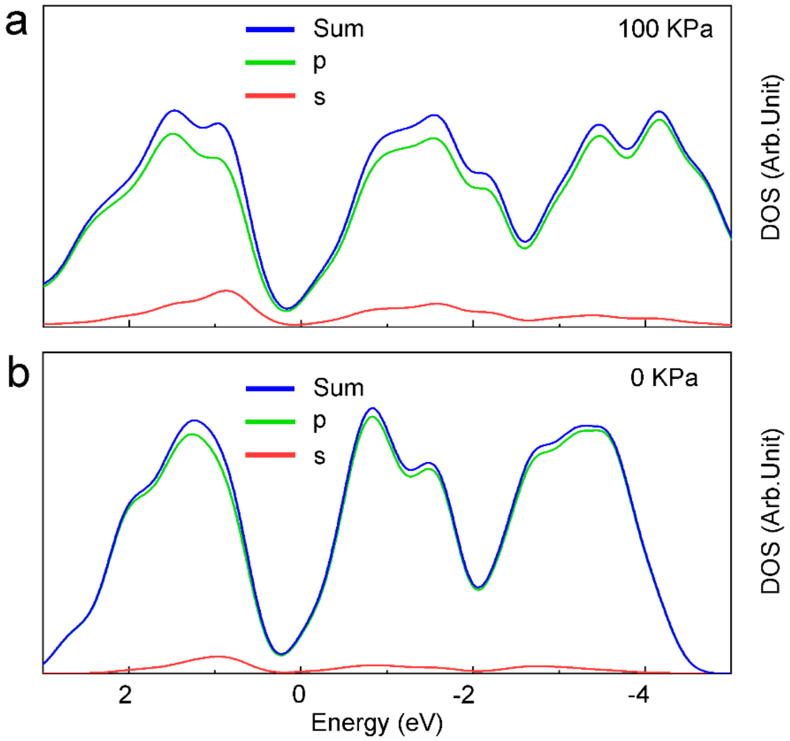

Supplementary Fig. 6 DOS of Te under different additional external pressure in addition to one atmosphere. DOS of Te under different additional external pressure in addition to one atmosphere: **a** 0 MPa; **b** 0.1 MPa along c-axis.

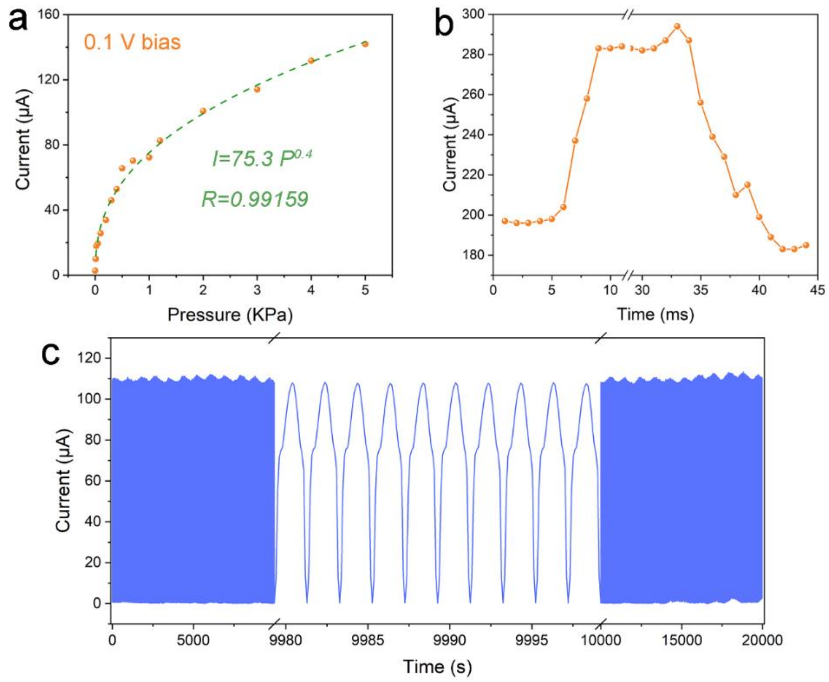

Supplementary Fig. 7 The piezoresistive properties characterization of single sensor. a The curve of the current to the pressure at 0.1 V. **b** The piezoresistive response time of the BTS. **c** The response current of 10000 cycles at 2 Hz.

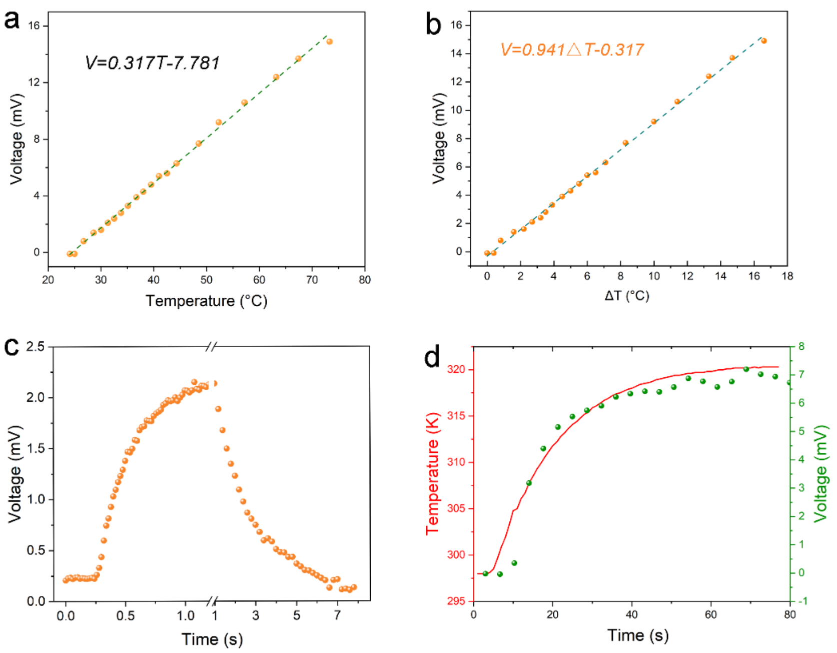

Supplementary Fig. 8 Thermoelectric property characterization of single sensor. a The curve of the voltage to bottom temperature. **b** The dependence of the measured output voltage on temperature difference from 0 K to 17 K. **c** The thermoelectric response time of the BTS. **d** The bottom temperature and output voltage as a function of time.

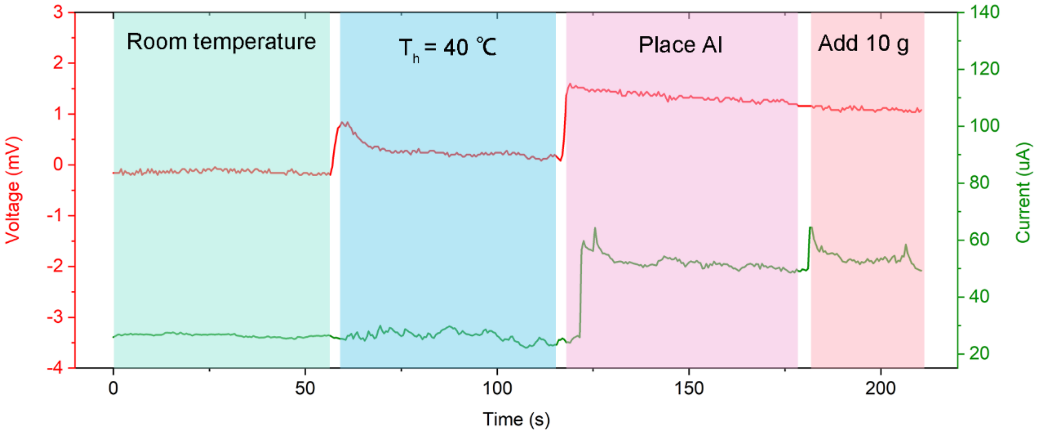

Supplementary Fig. 9 Thermoelectric and piezoresistive responses of single device to different materials. A real-time monitoring test of BTS under different external conditions: heating to 40 °C, aluminum block, 10 g of weight, which were applied in sequence.

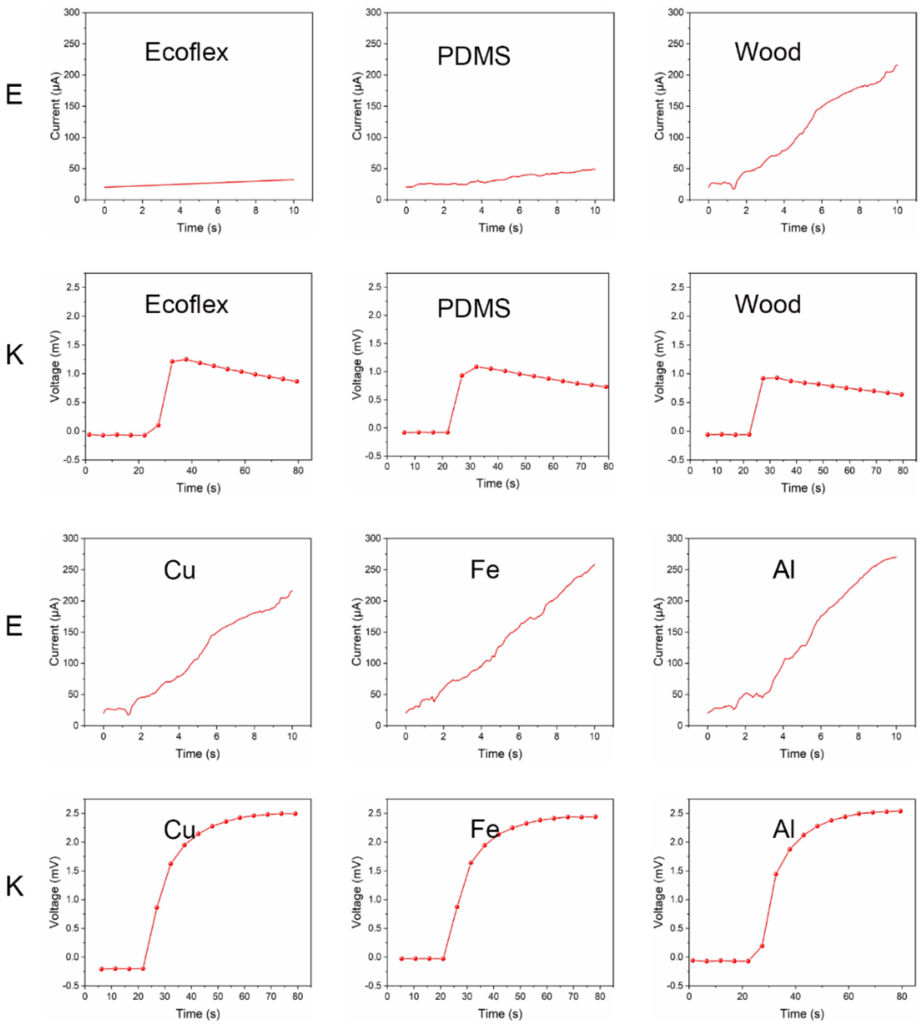

Supplementary Fig. 10 Thermoelectric and piezoresistive responses of single device to different materials. E: elastic modulus and K: thermal conductivity coefficient.

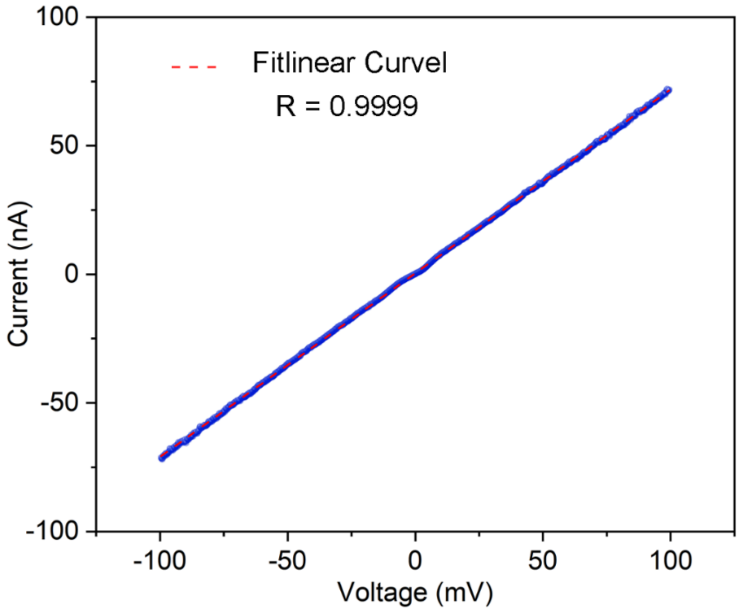

Supplementary Fig. 11 IV curve of the DTS under -100 mV - 100 mV bias. IV curve of the DTS under -100 mV - 100 mV bias and the fitlinear curve to it.

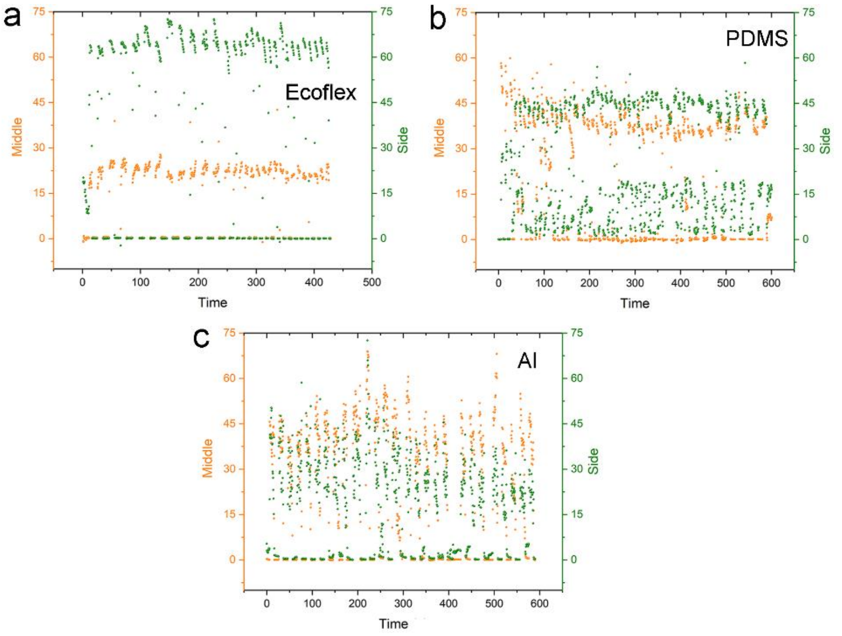

Supplementary Fig. 12 The H dataset. Unprocessed H dataset of (**a**) Ecoflex, (**b**) PDMS, (**c**) Al.

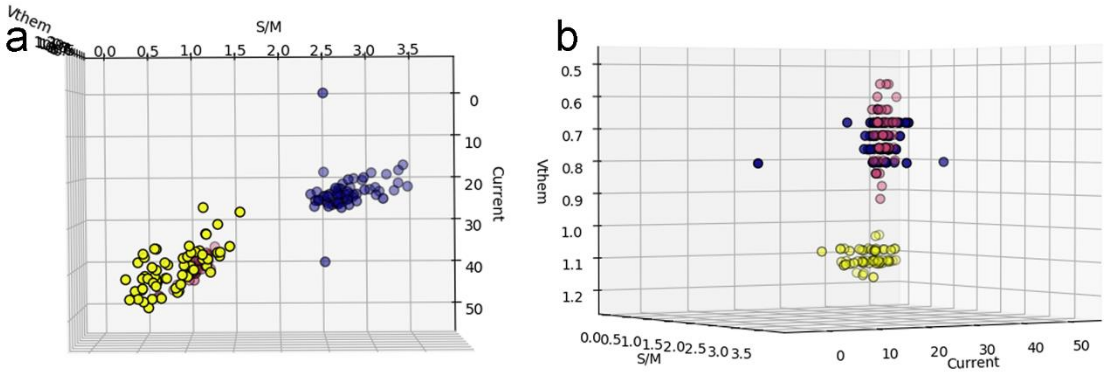

Supplementary Fig. 13 Distribution of sample points in one dimension. a Hardness. **b** Thermal conductivity.

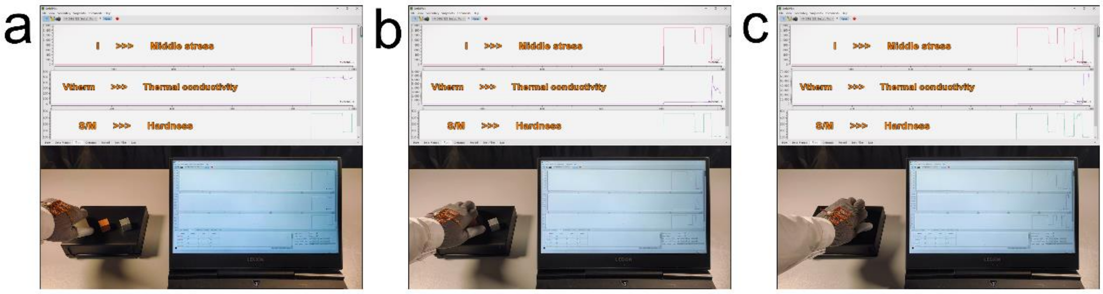

Supplementary Fig. 14 The process of identifying different materials. a Ecoflex. **b** Wood. **c** Al.

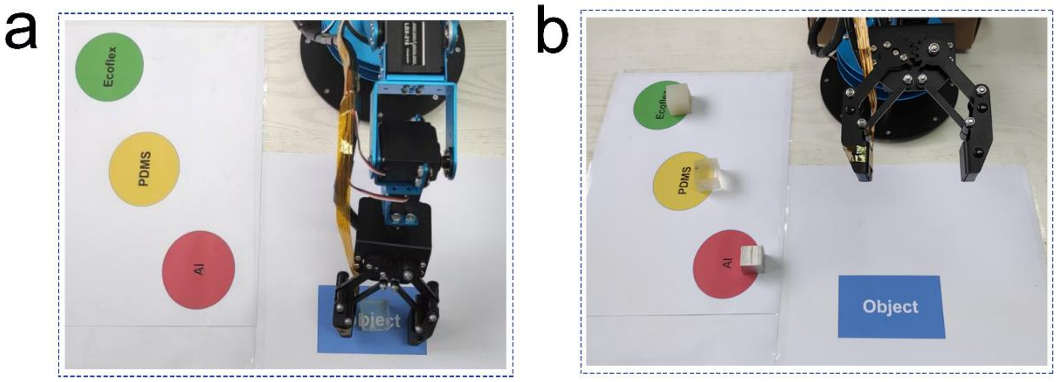

Supplementary Fig. 15 BTS is applied to the manipulator for material differentiation. a To be identified. **b** Identification completed.

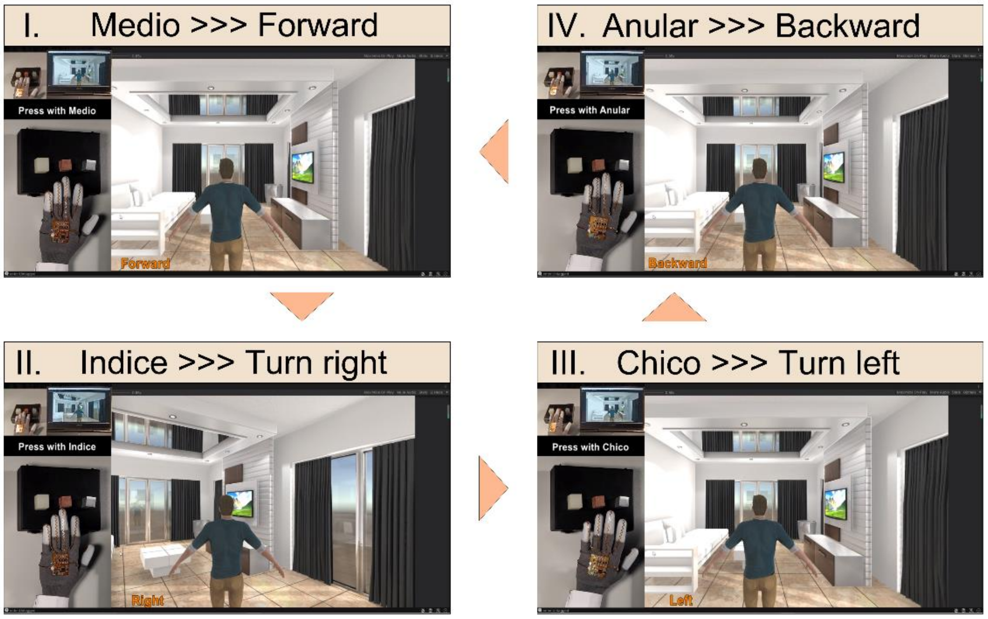

Supplementary Fig. 16 Movement control in VR world via smart glove. Do the following actions in sequence to direct the movement of the character: (I) press medio to forward; (II) press indice to turn right; (III) press chico to turn left; (IV) press anular to backward.

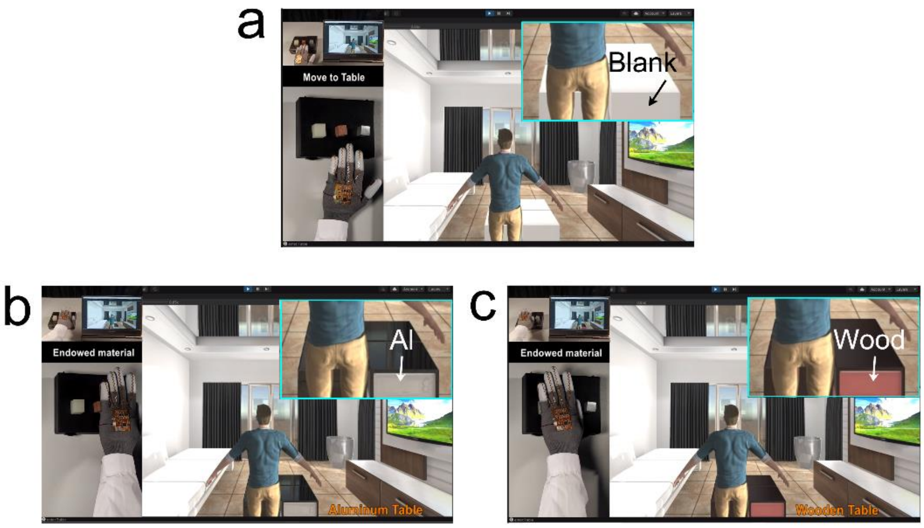

Supplementary Fig. 17 Endow a blank table with different materials. a a blank table. **b** Endow table with Al and get an Aluminum table. **c** Endow table with wood and get a wood table.

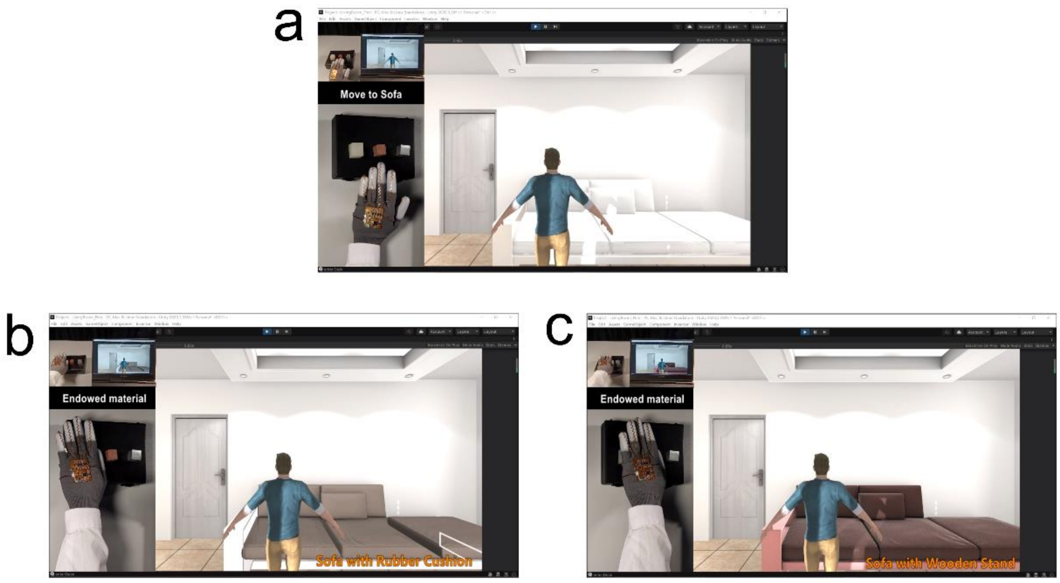

Supplementary Fig. 18 Give different parts of the sofa different materials. a a blank sofa. **b** sofa cushion rubber. **c** sofa stand wood.

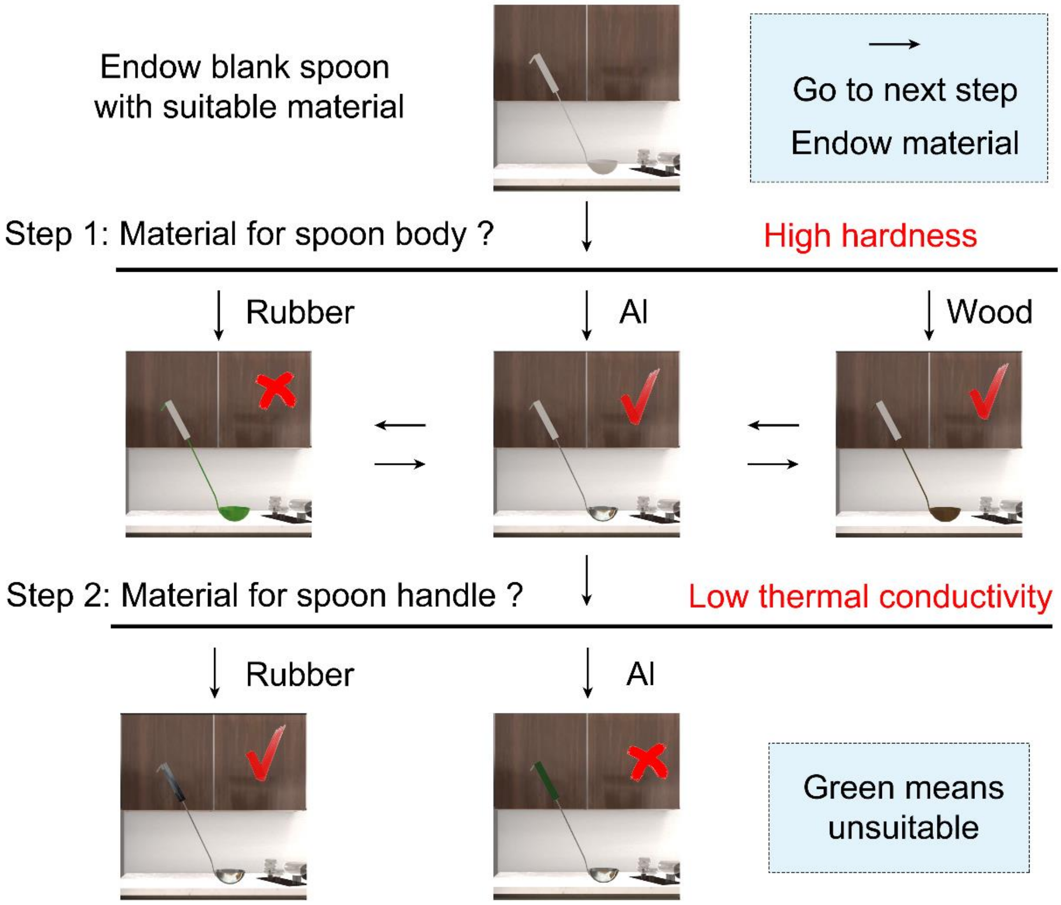

Supplementary Fig. 19 The logic diagram for assembling a spoon in a puzzle game. The game is played in one direction, that of the arrow. And these arrows indicate next step or assignment of materials. During the game, the spoon turns green to indicate that the material is unsuitable.

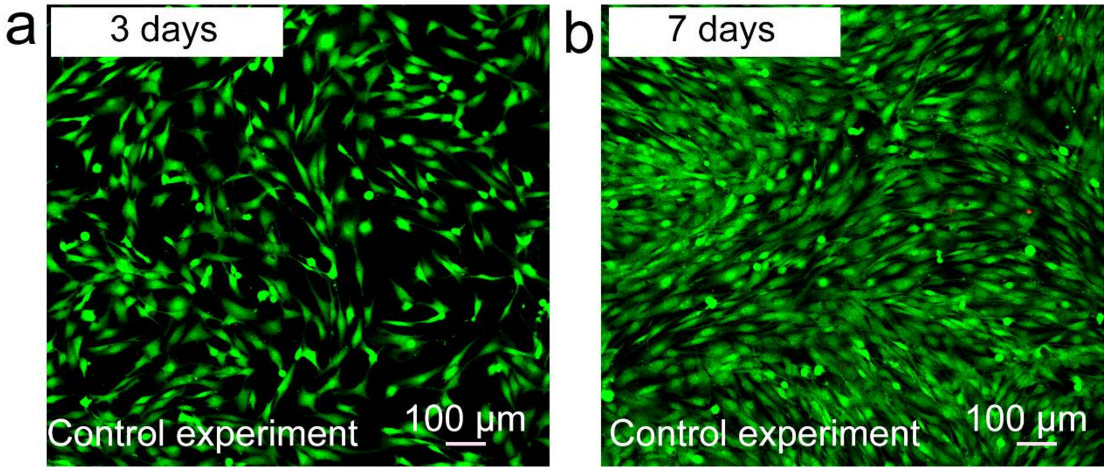

Supplementary Fig. 20 Fluorescence images of the human skin fibroblasts-HSF for 3 days and 7days control experiment. a 3days. **b** 7days.

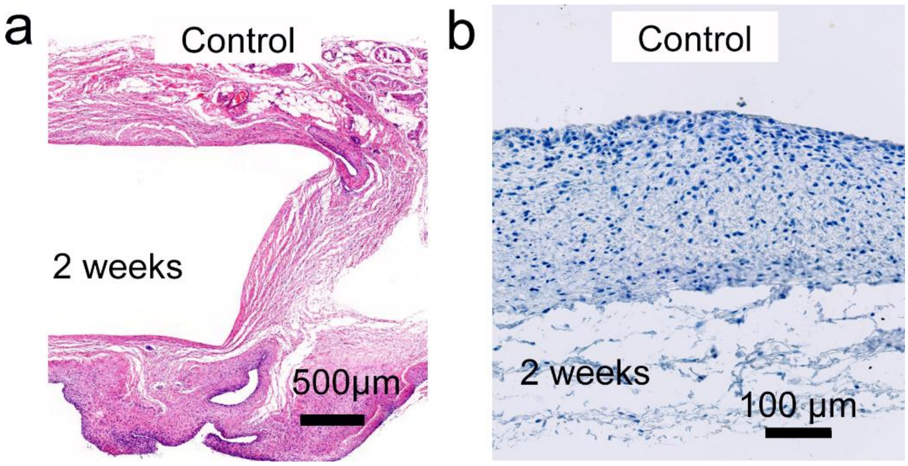

Supplementary Fig. 21 Results of biocompatibility assessment in vivo for control. a paraffin sections for H&E staining. **b** Frozen sections for immunohistochemistry.

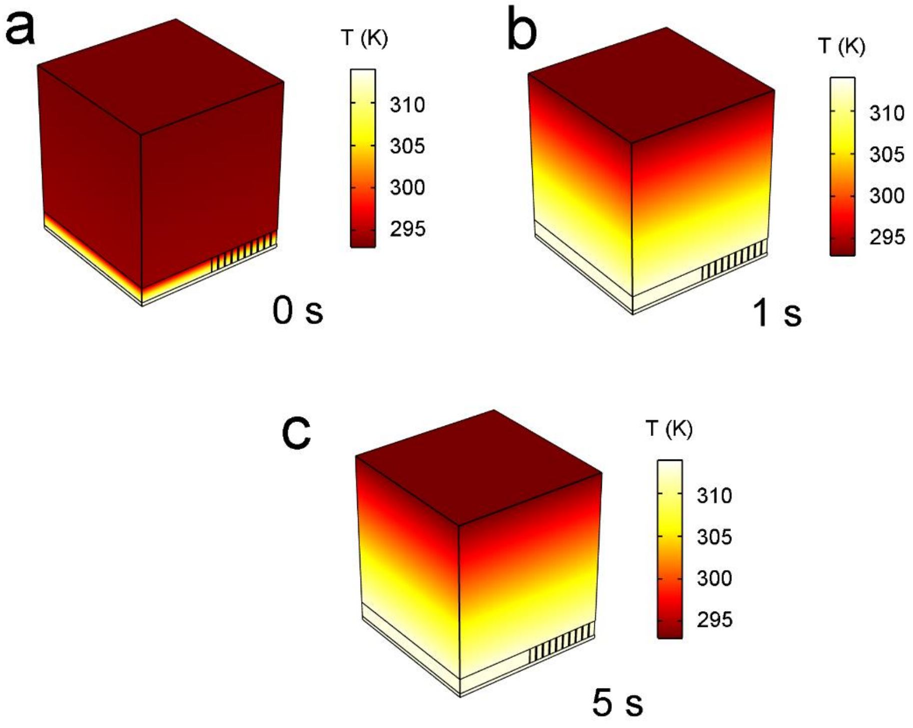

Supplementary Fig. 22 Temperature distribution at different time points. a 0 s, **b** 1 s, and **c** 5 s.

Supplementary Table 1 Hardness information of Material

Material	Ecoflex	PDMS	Wood	Al	Fe	Cu
Shore hardness (HD)	4	6.5	67	98.5	99.5	99.3

Supplementary Table 2 Functional comparisons between selected tactile sensors.

Ref.	Number in Functional Unit	P		T		Compatibility of output signal
		Active materials	Output signal	Active materials	Output signal	
1	2	Ag/PDMS	Voltage	Pt	Voltage	-
2	1	MOF-MSMC/PP	Current	MOF-MSMC/PP	Voltage	No
3	1	EMIM TFSI@Ag/SEBS	Impedance	EMIM TFSI@Ag/SEBS	Impedance	Yes
4	2	Ecoflex	Capacitance	Pt	Current	-
5	2	PDMS	Capacitance	rGO	Current	-
6	1	Ecoflex	Capacitance	Ecoflex	Capacitance	No
7	2	CNT	Current	Ag/MWCNT	Voltage	-
8	1	C-PDMS	Current	C-PDMS	Current	No
9	1	PU-PEDOT：PSS	Current	PU-PEDOT：PSS	Voltage	No
10	1	MXene-AgNW-PEDOT:PSS-TeNW	Current	MXene-AgNW-PEDOT:PSS-TeNW	Voltage	No
11	1	PEDOT:PSSW-PE	Current	PEDOT:PSSW-PE	Voltage	No
12	2	PVDF	Voltage	Te-MWCNT/PANI	Voltage	-
13	2	PDMS/silk carbon fiber (fractured)	Current	PDMS/silk carbon fiber (continuous)	Current	-
14	2	Ecoflex	Capacitance	Silk fiberearbon	Current	-
15	2	MXene/CNT	Current	MXene	Current	-
16	1	PEDOT:PSS/PDMS	Impedance	PEDOT:PSS/PDMS	Impedance	No
This work	1	Te	Current	Te	Current	Yes

Supplementary Table 3 Functional comparison of VR controller between selected research.

Ref.	Mechanism	Materials	Measured signal	Control mode	Application in VR	Year
17	Triboelectric	CNT/TPE coated textile	Bending	Finger bending	Motion control	2020
18	Piezoresistance	Commercial thin film	Bending & Pressure	Finger bending	Motion control	2022
19	Triboelectric	PEDOT:PSS coated textile	Bending	Finger bending	Mobile control	2019
20	Triboelectric	Ecoflex/wrinkled nitrile	Bending	Finger bending	Sign language translation	2021
21	Triboelectric	Galinstan-PDMS	Relative movement	Move positive material ball	Mobile control	2018
22	Triboelectric	Cu/FEP	Relative movement	Move material	Mobile control	2020
23	Triboelectric	Skin/PDMS-Ecoflex	Relative movement	Touch	Motion control	2020
24	Triboelectric	Nylon-polyester/stainless steel	Bending & Pressure	Finger bending	Motion control	2019
25	Piezoresistance	PU-AgNP fiber	Bending & Pressure	Finger bending & touch	Motion control	2020
26	Triboelectric	Nitrile/Ecoflex	Pressure	Walking	Motion control	2020
This work	Piezoresistance	Tellurium	Pressure & temperature difference	Touch & touch materials	Mobile control & Material giving	-

Supplementary Table 4 The specific material parameters in simulations

Device material	Au	Te1	Te2
Thermal conductivity ($Wm^{-1}K^{-1}$)	317	2.27	2.27
Electrical conductivity (S/m)	4.56E7	1.00E4	1.00E4
Relative permittivity	0	36	36
Seebeck coefficient (V/K)	0.0000065	0.0000065	0.001

Supplementary Note 1

During the experiment, it was found that there was no potential difference between the top and bottom of the NW on the gold-plated side of the device, and the resistance on this side was greatly reduced. Previous experiments can prove that tellurium tends to form compounds with electrode materials such as gold, silver, and copper.[27-29] Therefore, the tellurium nanowires on this side may form high-conductivity alloys with Au on the substrate. This can also be confirmed by the difference of the three XRD patterns in Supplementary Fig. 1. Considering that the thermoelectric potential is only related to the temperature difference and Seebeck coefficient, the NW on the gold-plated side is simplified as a block, and the Seebeck coefficient is the same as that of gold, so that it is not enough to generate an electric potential. Additionally, the Te NW has a thin film connection at the bottom; this film has a resistance of around 1000 ohms and no thermoelectric potential difference. This film may be thought of as the bottom electrode; hence, the bottom is referred to simply as the gold electrode layer.

Supplementary Note 2

The experimental results show that the relationship between current and displacement is approximately positively correlated, as shown in the Supplementary Fig. 11. As shown in the Figure 3a, the current of the three electrodes from left to right is defined as I_1, I_2, and I_3 with a bias voltage of 100 mV. As shown in Figure 3c, when the displacement is constant, the slope of the

curve can be mapped according to sides and intermediate current, which reflects the hardness information. The channel 1 in the user interface is I_2=I_1, reflecting the pressure. The channel 3 is S/M =(I_1+I_3)/2I_2. Because individual habitual forces are roughly the same, material hardness information can be directly inferred from S/M, and can be more accurately inferred by combining with I_2.

The channel 2 corresponds to V_{therm}. The intermediate current of the device is output as I_H under the applied bias voltage of 100mV. Without bias, the output is I_L. The time of the two voltage transitions was 20ms, during which the hand pressure did not change much. For two consecutive points (100 mV, I_H) and (0, I_L), the changes in device conductance are negligible and the two points can be regarded as being on the same IV curve, whose slope is the conductance at this point and intercept is the thermoelectric potential. Thermoelectric potential can be expressed as:

$$V_{therm} = \frac{100mV * I_L}{I_L - I_H}$$

where I_L is less than the 2.5% of I_H (Supplementary Fig. 11), which is relatively negligible. Therefore, the formula can be simplified as:

$$V_{therm} = \frac{100mV * I_L}{-I_H}$$

Supplementary Note 3

There is an error in the calculation of V_{therm}. As can be seen from equation

$$V_{therm} = \frac{100mV*I_L}{I_L-I_H} \qquad (1),$$

the calculated thermoelectric potential (V_{therm}) mainly depends on the ratio of

the currents at the two time points. If the low level is used at time point 1 and the high level is used at time point 2, then we can get

$$I_L = V_1 * k_1 \tag{2}$$

$$I_H = V_2 * k_2 \tag{3}$$

where V_1 and V_2 are the actual thermoelectric potentials at the two time points, and k_1 and k_2 are the actual conductance at the two time points. Substituting equations (2) and (3) into equation (1), we get

$$V_{therm} = \frac{100mV * V_1 k_1}{V_1 k_1 + V_2 k_2 - 100mV * k_2} \tag{4}$$

Equation 4 states that V_{therm} depends on the actual thermoelectric potential and conductance at both instants. In order to reduce interference to the calculation of V_{therm}, the following methods are adopted:

First, increase the sampling frequency. By reducing the sampling interval to 20ms, the changes in thermoelectric potential and internal resistance within the sampling interval are reduced. The effect of this method is more obvious for the relatively slow temperature response.

Second, artificially reduce the magnitude of pressure variation. When identifying materials, we usually hold the BTS firmly against the object for a few seconds. In this way, on the one hand, a stable signal is obtained, which is convenient for the BTS to make accurate distinctions; on the other hand, A few seconds to ensure that the temperature field between the BTS and the item stabilizes.

Third, reduce random errors by recursive mean sampling. When the

temperature field between the BTS and the item stabilizes, the actual thermoelectric potential of adjacent sampling points is approximately the same. Equation (4) can be simplified to

$$V_{therm} = \frac{100mV * Vk_1}{-100mV * k_2}$$

where V is actual thermoelectric potential. It can be seen that the error of V_{therm} is mainly caused by the finger pressure disturbance, and exists above and below the fraction. For such random errors, average sampling is used to reduce the interference caused by random errors. When judging the material, we set the material to be output when the same material is judged multiple times in a row, which further reduces the error.

Based on the above experimental methods, it could be ignored that the use of the internal resistance and thermoelectric voltage at different time points cause certain interference to the calculation of V_{therm}.

Supplementary Note 4

Since the detection of external temperature and pressure signals by units is the foundation of tactile sense. Analyzing a vast number of data sequences created by the array combination of individual units may also be used to recognize object form. As a result, the performance of functional components in the multimodal tactile system is compared and assessed here. It's clear from Number in Functional Unit that some of the study involves stacking numerous sensors rather than using a single sensing unit to detect various signals. On the other hand, while there are materials that can be used to make OTM devices,

there have been few experiments that have decoupled the signals of P and T. As a result, this research first picks Te, a multifunctional material, and then uses orientation design to complete the decoupling of input signals, laying the groundwork for OTM devices. Then, thanks to the acquisition circuit's design, the P and T output signals are consistent and do not interfere with one another, and the BTS in OTM mode is ready.

Supplementary References

1 Li G*, et al.* Skin-inspired quadruple tactile sensors integrated on a robot hand enable object recognition. *Sci. Robot.* **5**, eabc8134 (2020).

2 Li Y*, et al.* Mutually Noninterfering Flexible Pressure-Temperature Dual-Modal Sensors Based on Conductive Metal-Organic Framework for Electronic Skin. *ACS Nano* **16**, 473-484 (2021).

3 You I*, et al.* Artificial multimodal receptors based on ion relaxation dynamics. *Science* **370**, 961-965 (2020).

4 Hua Q*, et al.* Skin-inspired highly stretchable and conformable matrix networks for multifunctional sensing. *Nat. Commun.* **9**, 244 (2018).

5 Ho D H*, et al.* Stretchable and Multimodal All Graphene Electronic Skin. *Adv. Mater.* **28**, 2601-2608 (2016).

6 Kim S Y*, et al.* Highly Sensitive and Multimodal All-Carbon Skin Sensors Capable of Simultaneously Detecting Tactile and Biological Stimuli. *Adv. Mater.* **27**, 4178-4185 (2015).

7 Jung M*, et al.* Flexible multimodal sensor inspired by human skin based on hair-type flow, temperature, and pressure. *Flex. Print. Electron.* **5**, 025003 (2020).

8 Lee J H*, et al.* A Behavior-Learned Cross-Reactive Sensor Matrix for Intelligent Skin Perception. *Adv. Mater.* **32**, e2000969 (2020).

9 Zhang F*, et al.* Flexible and self-powered temperature-pressure dual-parameter sensors using microstructure-frame-supported organic

thermoelectric materials. *Nat. Commun.* **6**, 8356 (2015).

10 Li F*, et al.* Printable and Stretchable Temperature-Strain Dual-Sensing Nanocomposite with High Sensitivity and Perfect Stimulus Discriminability. *Nano Lett.* **20**, 6176-6184 (2020).

11 Han S*, et al.* Thermoelectric Polymer Aerogels for Pressure-Temperature Sensing Applications. *Adv. Funct. Mater.* **27**, 1703549 (2017).

12 Zhu P*, et al.* A flexible active dual-parameter sensor for sensitive temperature and physiological signal monitoring via integrating thermoelectric and piezoelectric conversion. *J. Mater. Chem. A* **7**, 8258-8267 (2019).

13 Wang C*, et al.* An All-Silk-Derived Dual-Mode E-skin for Simultaneous Temperature-Pressure Detection. *ACS Appl. Mater. Interfaces* **9**, 39484-39492 (2017).

14 Wu R*, et al.* Silk Composite Electronic Textile Sensor for High Space Precision 2D Combo Temperature-Pressure Sensing. *Small* **15**, e1901558 (2019).

15 Xue X*, et al.* Flexible Dual-Parameter Sensor Array without Coupling Based on Amorphous Indium Gallium Zinc Oxide Thin Film Transistors. *Adv. Mater. Technol.* **7**, 2100849 (2021).

16 Wen F*, et al.* Machine Learning Glove Using Self-Powered Conductive Superhydrophobic Triboelectric Textile for Gesture Recognition in

VR/AR Applications. *Adv. Sci.* **7**, 2000261 (2020).

17 Liu Y*, et al.* Electronic skin as wireless human-machine interfaces for robotic VR. *Sci. Adv.* **8**, eabl6700 (2022).

18 He T*, et al.* Self-powered glove-based intuitive interface for diversified control applications in real/cyber space. *Nano Energy* **58**, 641-651 (2019).

19 Wen F*, et al.* AI enabled sign language recognition and VR space bidirectional communication using triboelectric smart glove. *Nat. Commun.* **12**, 5378 (2021).

20 Chen T*, et al.* Novel augmented reality interface using a self-powered triboelectric based virtual reality 3D-control sensor. *Nano Energy* **51**, 162-172 (2018).

21 Hou C*, et al.* A Delta‐Parallel‐Inspired Human Machine Interface by Using Self-Powered Triboelectric Nanogenerator Toward 3D and VR/AR Manipulations. *Adv. Mater. Technol.* **6**, 2000912 (2020).

22 Zhu M*, et al.* Haptic-feedback smart glove as a creative human-machine interface (HMI) for virtual/augmented reality applications. *Sci. Adv.* **6**, eaaz8693 (2020).

23 He Q*, et al.* An all-textile triboelectric sensor for wearable teleoperated human–machine interaction. *J. Mater. Chem. A* **7**, 26804-26811 (2019).

24 Choi S*, et al.* Conductive Hierarchical Hairy Fibers for Highly Sensitive, Stretchable, and Water‐Resistant Multimodal Gesture‐Distinguishable

Sensor, VR Applications. *Adv. Funct. Mater.* **29**, 1905808 (2019).

25 Zhang Z*, et al.* Deep learning-enabled triboelectric smart socks for IoT-based gait analysis and VR applications. *npj Flex. Electron.* **4**, 1-12 (2020).

26 Lo L W*, et al.* A Soft Sponge Sensor for Multimodal Sensing and Distinguishing of Pressure, Strain, and Temperature. *ACS Appl. Mater. Interfaces* **14**, 9570-9578 (2022).

27 Bortz M L, *et al*. An investigation of the growth of Au and Cu on the van der waals surfaces of MoTe2 and WTe2. *Surf. Sci.* **223**, 285-298 (1989).

28 Hsieh H C, *et al*. Electroless Co-P diffusion barrier for n-PbTe thermoelectric material. *J. Alloy. Compd.* **728**, 1023-1029 (2017).

29 Qiu G, *et al*. Thermoelectric Performance of 2D Tellurium with Accumulation Contacts. *Nano Lett.* **19**, 1955-1962 (2019).
