# Supplementary material for: Dual sensing signal decoupling based on tellurium anisotropy for VR interaction and neuro-reflex system application
Source: Nat Commun. 2022 Oct 10;13:5975. doi: 10.1038/s41467-022-33716-9 (PMC9550802; doi:10.1038/s41467-022-33716-9)
Supplement: Supplementary file 2 — Description of Additional Supplementary Files [file 41467_2022_33716_MOESM2_ESM.pdf]

Description of Additional Supplementary Files

File Name: Supplementary Movie 1
Description: Differentiate the material by BTS.

File Name: Supplementary Movie 2
Description: The sensor on machinery for cognition.

File Name: Supplementary Movie 3
Description: Move in VR space.

File Name: Supplementary Movie 4
Description: Transform environments in VR space.

File Name: Supplementary Movie 5
Description: Endow material in VR space.

File Name: Supplementary Movie 6
Description: The sensor replaces rabbit skin for cognition.
