# Supplementary material for: Dual sensing signal decoupling based on tellurium anisotropy for VR interaction and neuro-reflex system application
Source: Nat Commun. 2022 Oct 10;13:5975. doi: 10.1038/s41467-022-33716-9 (PMC9550802; doi:10.1038/s41467-022-33716-9)
Supplement: Supplementary file 9 — Reporting Summary [file 41467_2022_33716_MOESM9_ESM.pdf]

nature portfolio

Corresponding author(s): Lili Wang, Guozhen Shen

Last updated by author(s): Sep 8, 2022

Reporting Summary

Nature Portfolio wishes to improve the reproducibility of the work that we publish. This form provides structure for consistency and transparency in reporting. For further information on Nature Portfolio policies, see our Editorial Policies and the Editorial Policy Checklist.

Statistics

For all statistical analyses, confirm that the following items are present in the figure legend, table legend, main text, or Methods section.

n/a	Confirmed	
☐	☒	The exact sample size (n) for each experimental group/condition, given as a discrete number and unit of measurement
☐	☒	A statement on whether measurements were taken from distinct samples or whether the same sample was measured repeatedly
☐	☒	The statistical test(s) used AND whether they are one- or two-sided<br>*Only common tests should be described solely by name; describe more complex techniques in the Methods section.*
☒	☐	A description of all covariates tested
☒	☐	A description of any assumptions or corrections, such as tests of normality and adjustment for multiple comparisons
☐	☒	A full description of the statistical parameters including central tendency (e.g. means) or other basic estimates (e.g. regression coefficient) AND variation (e.g. standard deviation) or associated estimates of uncertainty (e.g. confidence intervals)
☒	☐	For null hypothesis testing, the test statistic (e.g. F, t, r) with confidence intervals, effect sizes, degrees of freedom and P value noted<br>*Give P values as exact values whenever suitable.*
☒	☐	For Bayesian analysis, information on the choice of priors and Markov chain Monte Carlo settings
☒	☐	For hierarchical and complex designs, identification of the appropriate level for tests and full reporting of outcomes
☒	☐	Estimates of effect sizes (e.g. Cohen's d, Pearson's r), indicating how they were calculated

Our web collection on statistics for biologists contains articles on many of the points above.

Software and code

Policy information about availability of computer code

Data collection	Data were collected by Keithley B1500A, CHI 760E, UNI-T(UT325).
Data analysis	Data were analysed via Origin 2018 (64-bit), PyCharm Community Edition and Microsoft Excel 2021 (version 2207,15427.20210).

For manuscripts utilizing custom algorithms or software that are central to the research but not yet described in published literature, software must be made available to editors and reviewers. We strongly encourage code deposition in a community repository (e.g. GitHub). See the Nature Portfolio guidelines for submitting code & software for further information.

Data

Policy information about availability of data

All manuscripts must include a data availability statement. This statement should provide the following information, where applicable:

- Accession codes, unique identifiers, or web links for publicly available datasets
- A description of any restrictions on data availability
- For clinical datasets or third party data, please ensure that the statement adheres to our policy

The authors declare that all data supporting the findings of this study are available within the paper and its Supplementary Information. The raw and analysed datasets generated during the study are available from the corresponding authors on request.

Human research participants

Policy information about studies involving human research participants and Sex and Gender in Research.

Reporting on sex and gender	The study did not involve sex and gender.
Population characteristics	The study did not involve population characteristics.
Recruitment	The study did not involve recruitment.
Ethics oversight	Ethics oversight was not relevant to this study.

Note that full information on the approval of the study protocol must also be provided in the manuscript.

Field-specific reporting

Please select the one below that is the best fit for your research. If you are not sure, read the appropriate sections before making your selection.

☒ Life sciences ☐ Behavioural & social sciences ☐ Ecological, evolutionary & environmental sciences

For a reference copy of the document with all sections, see nature.com/documents/nr-reporting-summary-flat.pdf

Life sciences study design

All studies must disclose on these points even when the disclosure is negative.

Sample size	Sample sizes were not calculated beforehand. The sample size was determined by the number of biological and technical replicates necessary to convince us that the effect was real. We aimed for at least three biological replicates, and obtained several technical replicates for each sample.
Data exclusions	No data were excluded from the analyses.
Replication	All experimental findings were reliably reproduced.
Randomization	The experimental groups were formed based on what was being tested, with random selections.
Blinding	Blinding was not relevant to this study.

Reporting for specific materials, systems and methods

We require information from authors about some types of materials, experimental systems and methods used in many studies. Here, indicate whether each material, system or method listed is relevant to your study. If you are not sure if a list item applies to your research, read the appropriate section before selecting a response.

Materials & experimental systems

n/a	Involved in the study	
☐	☒	Antibodies
☐	☒	Eukaryotic cell lines
☒	☐	Palaeontology and archaeology
☐	☒	Animals and other organisms
☒	☐	Clinical data
☒	☐	Dual use research of concern

Methods

n/a	Involved in the study	
☒	☐	ChIP-seq
☒	☐	Flow cytometry
☒	☐	MRI-based neuroimaging

Antibodies

Antibodies used	KP1 to CD68 (abcam, ab955) used in this study are commercially available. Antibody validation was performed by the individual manufacturer and their data are available on the manufacturers' website. Please also see Methods for further description of antibodies, including dilutions used.
Validation	https://www.abcam.cn/CD68-antibody-KP1-ab955.html

Eukaryotic cell lines

Policy information about cell lines and Sex and Gender in Research

Cell line source(s)	HSF cell were obtained from icellbioscience (http://icellbioscience.com:8082/cellDetail/1905).
Authentication	The cell line is authenticated by STR method.
Mycoplasma contamination	All cell lines tested negative for mycoplasma contamination.
Commonly misidentified lines (See ICLAC register)	None of the used cell lines is listed in ICLAC database.

Animals and other research organisms

Policy information about studies involving animals; ARRIVE guidelines recommended for reporting animal research, and Sex and Gender in Research

Laboratory animals	Four-month-old New Zealand rabbit.
Wild animals	The study did not involve wild animals.
Reporting on sex	The study did not involve sex.
Field-collected samples	The study did not involve samples collected from the field.
Ethics oversight	All animal experiments were conducted in accordance with the regulations of Beijing Laboratory Animal Management Office (approval number: MDSW-2021-053C).

Note that full information on the approval of the study protocol must also be provided in the manuscript.
